# Vibrational-mechanical properties of the highly-mismatched Cd_1−x_Be_x_Te semiconductor alloy: experiment and ab initio calculations

**DOI:** 10.1038/s41598-023-39248-6

**Published:** 2023-09-04

**Authors:** A. Elmahjoubi, M. B. Shoker, O. Pagès, V. J. B. Torres, A. Polian, A. V. Postnikov, C. Bellin, K. Béneut, C. Gardiennet, G. Kervern, A. En Naciri, L. Broch, R. Hajj Hussein, J.-P. Itié, L. Nataf, S. Ravy, P. Franchetti, S. Diliberto, S. Michel, A. Abouais, K. Strzałkowski

**Affiliations:** 1 LCP-A2MC, UR 4632, Université de Lorraine, 57000 Metz, France; 2 Departamento de Fisica and i3N, Universidade de Aveiro, 3810 – 193 Aveiro, Portugal; 3 Institut de Minéralogie, de Physique des Matériaux et de Cosmochimie, Sorbonne Université — UMR CNRS 7590, 75005 Paris, France; 4 Synchrotron SOLEIL, L’Orme des Merisiers Saint-Aubin, BP 48, 91192 Gif-sur-Yvette Cedex, France; 5 Laboratoire de Cristallographie, Résonance Magnétique et Modélisations, CRM2, UMR 7036, Université de Lorraine, 54506 Vandoeuvre-lès-Nancy, France; 6 IJL, CNRS, Université de Lorraine, 57000 Metz, France; 7 Faculty of Physics, Astronomy and Informatics, Institute of Physics, Nicolaus Copernicus University in Toruń, Ul. Grudziądzka 5, 87-100 Toruń, Poland; 8 Engineering Science for Energy Lab, National School of Applied Sciences, Chouaib Doukkali University of El Jadida, El Jadida, Morocco; 9 Present Address: Department of Physics and Materials Science, University of Luxembourg, 41 rue du Brill, 4422 Belvaux, Luxembourg

**Keywords:** Materials science, Physics

## Abstract

The emerging CdTe–BeTe semiconductor alloy that exhibits a dramatic mismatch in bond covalency and bond stiffness clarifying its vibrational-mechanical properties is used as a benchmark to test the limits of the percolation model (PM) worked out to explain the complex Raman spectra of the related but less contrasted Zn_1−x_Be_x_-chalcogenides. The test is done by way of experiment ($x\le 0.11$), combining Raman scattering with X-ray diffraction at high pressure, and ab initio calculations ($x$ ~ 0–0.5; $x$~1). The (macroscopic) bulk modulus ${B}_{0}$ drops below the CdTe value on minor Be incorporation, at variance with a linear ${B}_{0}$ versus $x$ increase predicted ab initio, thus hinting at large anharmonic effects in the real crystal. Yet, no anomaly occurs at the (microscopic) bond scale as the regular bimodal PM-type Raman signal predicted ab initio for Be–Te in minority ($x$~0, 0.5) is barely detected experimentally. At large Be content ($x$~1), the same bimodal signal relaxes all the way down to inversion, an unprecedented case. However, specific pressure dependencies of the regular ($x$~0, 0.5) and inverted ($x$~1) Be–Te Raman doublets are in line with the predictions of the PM. Hence, the PM applies as such to Cd_1−x_Be_x_Te without further refinement, albeit in a “relaxed” form. This enhances the model’s validity as a generic descriptor of phonons in alloys.

## Introduction

Due to its direct optical band gap of 1.5 eV and high optical absorption coefficient, the cubic-zincblende II–VI CdTe semiconductor compound is almost ideal for solar energy conversion. The CdTe-based technology, stimulated by high conversion rates achieved over the past decade, exceeding 22% in laboratory, together with the stability of the photovoltaic devices under operating conditions, has now developed up to the industrial scale^1^. As early as in 1960’s, the alloying of CdTe has been explored as a convenient means to finely tune its physical properties being of major interest as those of a semiconductor, namely the optical band gap ${E}_{0}$ and the lattice constant $a$, in view of targeted applications. The current study of the prospective Cd_1−x_Be_x_Te II–VI semiconductor alloy is in this line. Yet, the focus is on the mechanical-vibrational properties; the optical (${E}_{0}$) and structural ($a$) ones are treated to a lesser extent.

One conventionally refers to II–VI and III–V pseudobinary A_1−x_B_x_C semiconductor alloys of first generation, still actively studied^2^, as those in which the A $\leftrightarrow$ B substitution involves elements from the third to sixth rows of the periodic table. The A–C and B–C chemical bonds enter with physical properties, notably length $l$ and covalency ${\alpha }_{c}$, close within a few percent. This facilitates alloying. The CdTe-based Cd_1−x_Zn_x_Te, CdSe_x_Te_1−x_ and CdS_x_Te_1−x_ systems are like this, forming “well-matched” alloys (WMA’s)^1^. In the exemplary III–V WMA, Al_1−x_Ga_x_As ($\frac{\Delta l}{l}\sim 8\permil$, $\frac{\Delta {\alpha }_{c}^{3}}{{\alpha }_{c}^{3}}\sim 5\%$), and the leading II–VI WMA, Cd_1−x_Hg_x_Te ($\frac{\Delta l}{l}\sim 3\permil$, $\frac{\Delta {\alpha }_{c}^{3}}{{\alpha }_{c}^{3}}\sim 2.5\%$), the matching is nearly perfect. Generally, the reported $l$ and ${\alpha }_{c}$ values throughout this work, given cubed in the latter case, are cited from Refs.^3,4^ unless specified.

In the 1990’s, the emergence of semiconductor alloys involving second-row elements in substitution like N, Be and O, e.g., N-dilute GaAs_1−x_N_x_ (Ref.^5^) and Zn_1−x_Be_x_-chalcogenides^6,7^, created a disruption. In fact, such light elements with small covalent radii form extremely short bonds. Moreover, the latter exhibit odd features of the ${\alpha }_{c}$ that governs the resistance of a bond to a distortion in shear (increasing with ${\alpha }_{c}$) and hence the stability of the lattice. So, with Be-bonding (BeS, BeSe, BeTe) the ${\alpha }_{c}$ achieves maximum among II–VI’s, whereas, on the contrary, the ${\alpha }_{c}$ of N-bonded compounds (InN, GaN, AlN) hits a minimum among III–V’s^4^. Consequently, in zincblende alloys such as Zn_1−x_Be_x_Se and GaAs_1−x_N_x_, the bond properties do dramatically differ, by as much as ($\frac{\Delta l}{l}\sim 9\%,$ $\frac{\Delta {\alpha }_{c}^{3}}{{\alpha }_{c}^{3}}\sim 33\%$) and ($\sim 20\%,$ $\sim 23\%$), respectively. The discrepancy softens as N is replaced by next-row P element to form GaAs_1−x_P_x_ ($\sim 3.5\%,$ $\sim 1\%$). The “bond mismatch” is likewise large for ZnSe_1−x_O_x_ ($\sim 21\%$—Ref.^8^, $\sim 16\%$) but small for ZnSe_1−x_S_x_ ($\sim 4.5\%$, $\sim 6.8\%$). Hence, the second-generation alloys involving Be, N and O in substitution were singled out as forming a new class of “highly-mismatched alloys” (HMA’s for short)^9^.

HMA’s, to which belongs Cd_1−x_Be_x_Te studied in this work, attract attention because in certain cases their large bond mismatch dramatically impacts the electronic band structure. For instance, even slight N-incorporation into GaAs, anyway limited to a few percent^10^, induces a giant negative bowing of ${E}_{0}$, i.e., of ~ 100 meV per atomic percent of N^10^, due to a band anticrossing. The latter results from coupling of the extended states forming the host conduction band with a quasi-resonant highly localized impurity state^10–14^. Oxygen likewise induces an intermediate band within the bandgap of O-dilute ZnTe_1−x_O_x_^15,16^ and CdTe_1−x_O_x_^17^. No similar outstanding features were detected with Zn_1−x_Be_x_-chalcogenides. Hence, the latter systems received less attention so far. ${E}_{0}$ varies linearly with $x$ in Zn_1−x_Be_x_Te^18^, or undergoes just a slight bowing in Zn_1−x_Be_x_Se^19^. More generally, the quasi linearity with composition governs all studied critical points of the electronic band structure of Zn_1−x_Be_x_-chalcogenides. We mainly refer to the direct gaps between the upper valence band and the lower conduction band at the $\Gamma$, $L$ and $X$ points, namely ${E}_{0}$, ${E}_{1}$ and ${E}_{2}$, respectively, and to the corresponding gaps involving the light hole valence band, notably ${E}_{0}+{\Delta }_{0}$ and ${E}_{1}+{\Delta }_{1}$, of both Zn_1−x_Be_x_Te^21^ and Zn_1−x_Be_x_Se^22^, as assigned in Ref.^20^.

However, the Zn_1−x_Be_x_-chalcogenides have held a central place in what regards vibrational properties. In early 2000’s, pioneering Raman studies^23–25^ revealed a bimodal signal per bond (1-bond $\to$ 2-mode) that contested the classical view of a unique mode per bond (1-bond $\to$ 1-mode) in random zincblende alloys^26^. This was explained in terms of sensitivity of the effective bond force constant $k$, probed by Raman scattering, to local Be- and Zn-like environments, casted into the percolation model (PM, Ref.^27^ and refs. therein). Subsequently, the PM enabled a unified understanding, hitherto missing, of the Raman spectra of II–VI, III–V and IV WMA’s with zincblende and diamond structures, suggesting the model’s universality^27^.

In this work, the PM is tested on the emerging cubic-Cd_1−x_Be_x_Te HMA that exhibits an even larger bond mismatch ($\frac{\Delta l}{l}\sim 15\%$, $\frac{\Delta {\alpha }_{c}^{3}}{{\alpha }_{c}^{3}}\sim 36\%$) than the Zn_1−x_Be_x_-chalcogenides. It is not a priori obvious whether the PM would apply to such special system. In the positive case, it will be instructive to elaborate on specific consequences from the PM, listed below from basic to more advanced ones^28^:

1. Only Be–Te should exhibit a distinct Raman doublet. Being small, Be has more room than Cd to move around in the Te-cage to accommodate the local strain created by the bond mismatch. Hence, the Raman frequencies are more diversified for Be–Te than for Cd–Te.
2. Out of the two sub-modes within the Be–Te doublet, the softer (resp. harder) one would refer to Be (resp. Cd)-like environments. Consider, *e.g.*, an isolated Cd atom embedded in BeTe. In its vicinity, the short Be–Te bonds suffer a compressive strain due to a competition with the longer Cd–Te ones within the given lattice spacing. Hence, they vibrate at a higher frequency than the bulk-like Be–Te bonds away from Cd. An inverse argumentation applies to CdTe doped with Be.
3. The Be–Te doublet is expected to converge under pressure. This is due to a larger volume derivative of the bond ionicity $\partial {f}_{i}/\partial lnV$, with ${f}_{i}=1-{\alpha }_{c}$, for Be–Te (0.7453) than for Cd–Te (0.407)^3^. Hence, the Be-like environment hardens under pressure faster than the Cd-like environment does. Accordingly, the related softer Be–Te Raman line within the percolation doublet drifts upwards under pressure faster. A classification of II–VI and III–V alloys, covering WMA’s and HMA’s, was accordingly suggested in Ref.^27^.
4. The convergence process in question either ends up into a phonon exceptional point at the crossing-resonance (scenario 1), or freely develops into an inverted doublet post resonance (scenario 2). This depends on whether the bond responsible for the vibration doublet is dispersed, *i.e*., self-connects in chains, or matrix-like, i.e., self-connects in bulk, respectively. The Raman intensity of the minor mode within the doublet undergoes, correspondingly, an extinction (scenario 1) or enhancement (scenario 2).

More generally, we aim at an integrated and coherent fundamental study of mechanical properties of the Cd_1−x_Be_x_Te HMA at the macroscopic and microscopic scales via elasticity and via the effective bond force constants, respectively. Zn_1−x_Be_x_Te^28^ is used as a suitable reference throughout the study based on proximity of Cd and Zn in the periodic table. Specifically, we combine powder high-pressure X-ray diffraction (HP-XRD) measurements at the PSICHÉ and CRISTAL beamlines of SOLEIL synchrotron, in search for the macroscopic bulk modulus ${B}_{0}$, with high-pressure Raman scattering (HP-RS) measurements on single crystals, probing the effective bond force constants in line with the above raised issues 1-to-4 around the PM. The discussion of Cd_1−x_Be_x_Te experimental data is supported by high-pressure ab initio snapshots of the lattice relaxation, notably to determine the equation of state from which ${B}_{0}$ is issued, and of the lattice dynamics, with special attention to the Raman frequencies and intensities. Additional ab initio calculations are implemented to cover $x$ values beyond the experimental range, currently limited to 11 at.% Be. Various ab initio codes are used, i.e., AIMPRO^29,30^ (Ab Initio Modeling PROgram), SIESTA^31,32^ and QE^33^ (Quantum Expresso), depending on need, as specified in the course of the discussion.

Besides, we briefly test by ellipsometry and transmission if the ${E}_{0}$ versus $x$ dependency is quasi linear, like with Zn_1−x_Be_x_-HMA’s, or significantly deviates from linearity, like with N- and O-dilute HMA’s. It is a matter to appreciate on an experimental basis whether HMAlloying with the second-row elements Be, N and O is virtuous for ${E}_{0}$, in that it generates a smooth linear-like ${E}_{0}$ versus $x$ variation, only in case of Zn $\leftrightarrow$ Be substitution, as discussed above, for whatever reason, or is a more general rule with Be.

## Results and discussion

**Figure 1:**
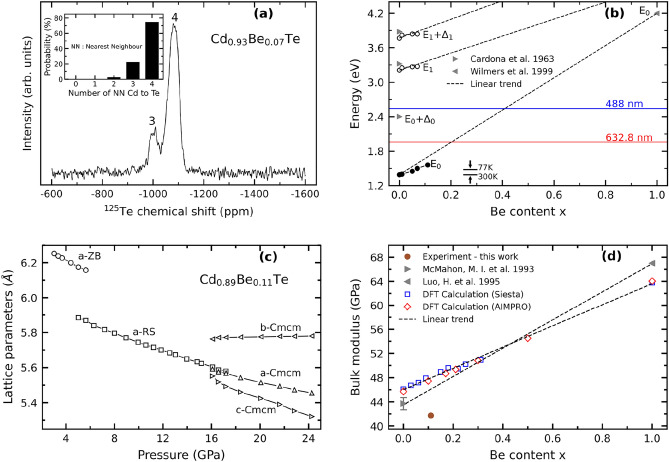
Cd_1−x_Be_x_Te structural, optical and mechanical properties. (**a**) CPMG Cd_0.93_Be_0.07_Te ^125^Te NMR signal. The binomial distribution of Te-centered nearest-neighbor (NN) tetrahedon clusters depending on the number of Cd atoms at the vertices in case of a random Cd $\leftrightarrow$ Be substitution is added for comparison (inset). The NMR peaks are labeled accordingly. (**b**) Composition dependence of the main Cd_1−x_Be_x_Te electronic transitions measured at room temperature by transmission (filled symbols, Fig. S5a) and ellipsometry (hollow symbols, Fig. S5b). CdTe (Ref.^37^) and BeTe (Ref.^20^) values taken from the literature are added, for reference purpose. Linear (dashed) trends between parent values are guidelines for the eye. Laser lines used to excite the Raman spectra are positioned to appreciate resonance conditions. Antagonist arrows help to appreciate the shift of electronic transitions by lowering temperature from ambient to liquid nitrogen, by referring to the ${E}_{0}$ gap of CdTe Ref.^38^. (**c**) Pressure dependence of the zincblende (zb), rocksalt (rs) and Cmcm (cm) Cd_0.89_Be_0.11_Te lattice constant(s) measured by high-pressure X-ray diffraction (Fig. S1c). (d) The ${B}_{0}$ value derived for Cd_0.89_Be_0.11_Te in its native zb phase (filled circle) from the corresponding volume vs. pressure dependence (Fig. S1d) is compared with the parent values taken from the literature (filled triangles, Refs.^39,40^) and with current ab initio data obtained with the AIMPRO (hollow diamonds) and SIESTA (hollow squares) codes. Corresponding linear $x$-dependencies are shown (dashed lines), for reference purpose.

The studied samples consist of high quality Cd_1−x_Be_x_Te and Zn_1−x_Be_x_Te bulk single crystals grown by the Bridgman method with small Be content, i.e., $x\le 0.11$ and 0.21, as determined by chemical analysis (see methods) and via the $a$ versus $x$ linearity^34^, correspondingly. The zincblende structure, common to end compounds, was confirmed by powder X-ray diffraction (Figs. S1 and S2; “S” stands for Supplementary Information) throughout intermediate compositions. The Cd_1−x_Be_x_Te lattice shrinks homothetically as $x$ increases, and inversely, reflected by a linear $a$ versus $x$ variation between the CdTe (this work) and BeTe^35^ values (Fig. S1b). This seems to be common in alloys, HMA’s included^2^. Further structural insight at the microscopic scale is gained via powder ($x$=0.07) ^125^Te solid-state nuclear magnetic resonance (NMR) measurements^36^. A bimodal NMR pattern (Fig. 1a) distinguishes between two tetrahedral environments for Te among five possible ones depending on the number of Cd and Be nearest neighbors. The NMR peak intensities scale as the fractions of tetrahedral Te-clusters with 4 × Cd and (3 × Cd, 1 × Be) atoms at the vertices as estimated from the Bernoulli’s binomial distribution^36^ at $x=0.07$ (Fig. 1a, inset). One notes a practical absence of Te-centered clusters with more Be atoms, consistently with experiment. Altogether the NMR data point towards an ideally random Cd ↔ Be substitution in Cd_0.93_Be_0.07_Te. The random substitution is presumably valid in all studied samples owing to the close Be contents. Hence the discussed experimental trends hereafter are intrinsic to random Cd_1−x_Be_x_Te-alloying. Additional (^9^Be and ^117^Cd) NMR experiment completing the current (^125^Te) NMR insight into Cd_0.93_Be_0.07_Te are reported in Fig. S4.

The Tauc plots of Cd_1−x_Be_x_Te transmission data ($x\le 0.11$, Fig. S5a) reveal a quasi linear ${E}_{0}$ vs. $x$ trend between the CdTe^37,38^ and BeTe^20^ values (Fig. 1b). The linearity persists with ${E}_{1}$ and ${E}_{1}+{\Delta }_{1}$ (Fig. 1b) accessed by ellipsometry (Fig. S5b), and also with ${E}_{2}$ (not shown) notwithstanding a poor signal-to-noise ratio due to the lack of luminous flux at high energy with the light source used. This resembles the case of Zn_1−x_Be_x_-chalcogenides^18–22^, contrasting that of dilute nitrides/oxydes^10–17^. Hence, the ${E}_{0}$ versus $x$ linearity seems to be the rule with Be substitution. The (Be, Zn, Cd) substituents involved in (Zn,Cd)_1−x_Be_x_-chalcogenides are nearly iso-electronegative (within few percent). In contrast, dilute nitrides and oxydes exhibit a large contrast in electronegativity between alloying elements (in the range 25–40%), the admitted cause^16^ for their large negative ${E}_{0}$ versus $x$ bowing^12–16^. This explains why the (Zn,Cd)_1−x_Be_x_-HMA’s behave like WMA’s in what regards their optical properties.

We turn now to the Cd_1−x_Be_x_Te mechanical-vibrational properties. These are successively addressed at the macroscopic scale at SOLEIL synchrotron (PSICHÉ beamline) searching for the bulk modulus ${B}_{0}$ by HP-XRD and at the microscopic scale by HP-RS, sensitive to the bond force constants, in relation to the raised issues 1-to-4 around the PM. Both studies are performed at the same Be content ($x=0.11$), for the sake of consistency.

The sequence of pressure-induced structural transitions (Fig. 1c) apparent in the Cd_0.89_Be_0.11_Te HP-XRD diffractograms taken on the upstroke (Fig. S1c) repeats that of pure CdTe (0 GPa: zincblende/ZB-cubic; 4 GPa: Rocksalt/RS-cubic, 12 GPa: Cmcm-orthorhombic)^39^. Only, the critical pressures are shifted to higher pressures (ZB $\to$ RS: 5 GPa, RS $\to$ Cmcm: 16 GPa) due to a reinforcement of the soft-ionic CdTe-lattice by the stiff-covalent Be-bonding. Such phenomenon came about also in Zn_1−x_Be_x_Se^40^ and Zn_1−x_Be_x_Te^28^. One disconcerting feature, though, is that ${B}_{0}$ derived for Cd_0.89_Be_0.11_Te (41.667 $\pm$ 0.243 GPa; the error bar is within the symbol size in Fig. 1d) from best fitting of the experimental pressure versus volume dependence via the Birch-Murnaghan equation^41^ drops below the ${B}_{0}$ value of CdTe^42^ (43.7 $\pm$ 1.0 GPa), which in turn is well below the BeTe one^43^ (~ 67 GPa). The latter parent values are added in Fig. 1d, for a direct comparison.

The ${B}_{0}$-drop is not accidental, due to the quality of fit. The first-order pressure derivative of ${B}_{0}$ evaluated at 0 GPa, ${B}_{0}{\prime}$, coming out in the fit is 4.00 $\pm$ 0.15 for Cd_0.89_Be_0.11_Te, nearly matching the CdTe^42^ value, i.e., 3.8 $\pm$ 0.6, and the BeTe one, fixed to 4 in Ref.^43^. By adopting for ${B}_{0}{\prime}$ the current “optimized” AIMPRO (${B}_{0}{\prime}=4.7$, ${B}_{0}=40.5$ GPa) and SIESTA (${B}_{0}{\prime}=5.1$, ${B}_{0}=39.7$ GPa) values at ~ 10 at.% Be (see below), the ${B}_{0}$-drop for Cd_0.89_Be_0.11_Te is emphasized and the quality of fit degraded. A similar ${B}_{0}$-drop is also evidenced by HP-XRD diffraction with Zn_1−x_Be_x_Te (Fig. S3b, $x\le 0.21$) using ${B}_{0}{\prime}=4$ for the fitting (Fig. S3a), that was found relevant for both ZnTe^44^ and BeTe^43^. Hence, the ${B}_{0}$-drop is apparently a common feature of BeTe-based alloys.

The ${B}_{0}$-drop deviates from the quasi linear ${B}_{0}$ versus $x$ experimental trend observed with Zn_1−x_Be_x_Se, however, disrupted by a punctual lattice hardening on percolation of the stiff Be–Se bonds^40^. The reason why Cd_1−x_Be_x_Te and Zn_1−x_Be_x_Te, but not Zn_1−x_Be_x_Se, suffer a ${B}_{0}$-drop from minor Be incorporation, might relate to a much weaker ionicity of the Be–Te chemical bonding (${f}_{i}$= 0.222) compared with Be-Se (${f}_{i}=0.420$)^3^. This gives rise to more dramatic $\Delta {f}_{i}/{f}_{i}$-contrasts in the BeTe-based Cd_1−x_Be_x_Te ($\sim$ 70%) and Zn_1−x_Be_x_Te ($\sim$ 60%) alloys than in the BeSe-based Zn_1−x_Be_x_Se one ($\sim$ 43%)^3^. This results in more severe bond distortions eventually affecting the mechanical properties in bulk.

No such ${B}_{0}$-drop but a linear ${B}_{0}$ versus $x$ trend is apparent in ab initio results ($x\le 0.5$, hollow symbols, Fig. 1d). The linearity seems robust since it is verified by two independent sets of calculations, applying the SIESTA and AIMPRO codes to distinct sets of fully- and partially-relaxed 64-atom (squares) and 216-atom (diamonds) quasirandom pseudobinary atomic arrangements (see methods), respectively. The ab initio versus experiment discrepancy around ${B}_{0}$ suggests large anharmonic effects in real Cd_1−x_Be_x_Te, developing on large length scales, i.e., beyond the finite supercell-sizes used for the current ab initio calculations. More precisely, the inability of the current ab initio calculations to reproduce the ${B}_{0}$-drop in either 64-atom or 216-atom disordered supercells hints that this ${B}_{0}$-drop does not find its origin in the random atom arrangement at the microscopic scale but rather at the mesoscopic scale in the relative topology of the (minor) BeTe-like and (dominant) CdTe-like environments. A decisive test to validate this hypothesis would be to perform ab initio calculations on disordered supercells containing thousands of substituent atoms, that would naturally involve the complexity of the mesostructure of a real randomly-disordered alloy. In this case, the random substitution can be simulated via special quasirandom structures designed in the spirit of Zunger et al.^45^, reproducing correlations up to a very large length scale^46^, and not limited to first-neighbors as in our case.

Now we address the microscopic vibrational properties of Cd_1−x_Be_x_Te, in line with the PM-related issues 1-to-4 around the Be-Te Raman signal raised in the introductory Section. A comparison with the reference Be–Te Raman percolation doublet of Zn_0.89_Be_0.11_Te^28^, matched in Be content with Cd_0.89_Be_0.11_Te, helps to fix ideas. Two impurity modes are involved in this case, a lower/minor ${TO}_{Be-Te}^{Be}$ and an upper/dominant ${TO}_{Be-Te}^{Zn}$. The subscript and superscript refer to the vibrating bond and to its local environment, respectively. The two modes are separated by ${\Delta }_{Be-Te}\sim 20$ cm^-1^ and exhibit Raman intensities differing by roughly an order of magnitude.

**Figure 2:**
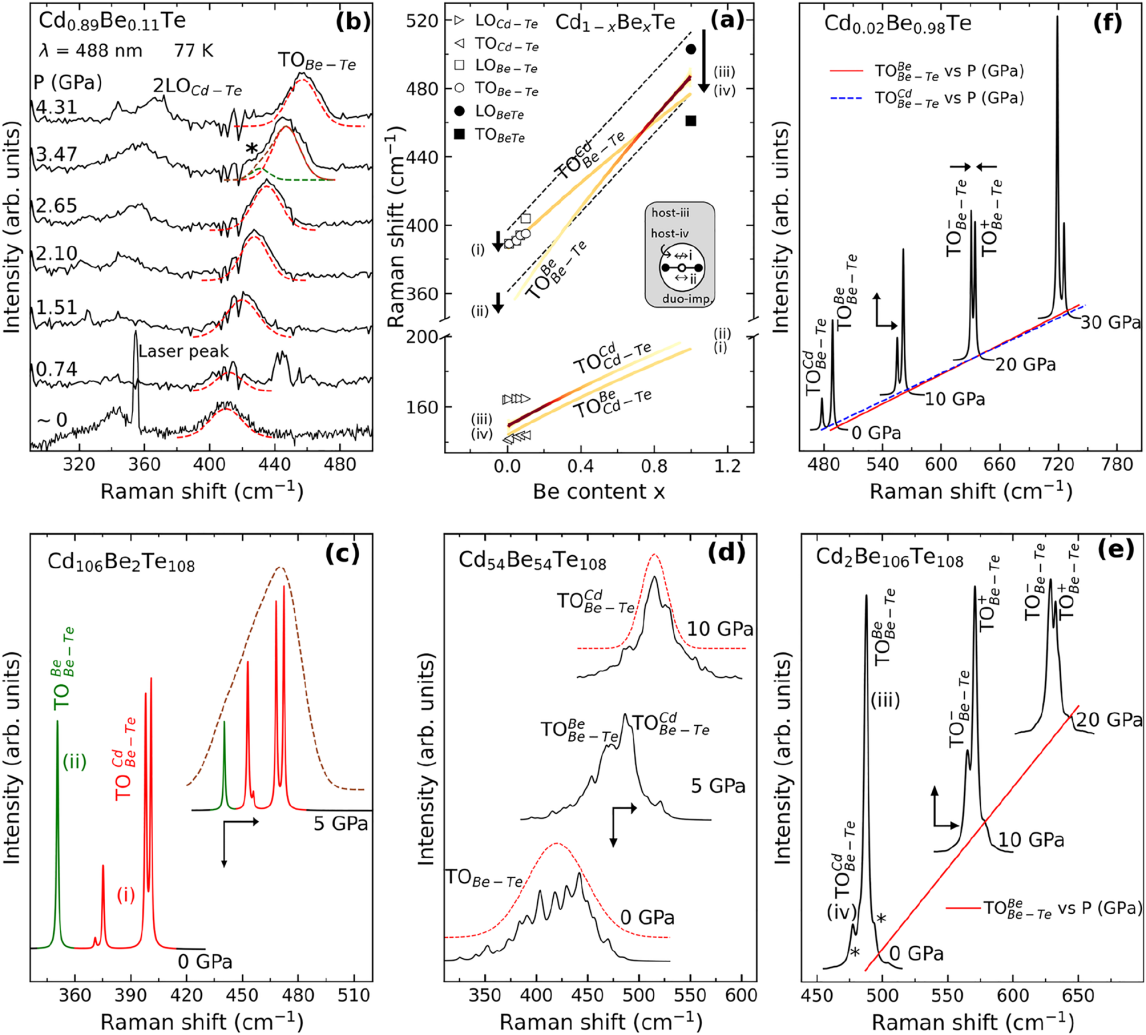
Cd_1−x_Be_x_Te vibrational properties. Panels are arranged anti-clockwise, with an overview at the top-center (**a**), in the sense of increasing $x$ values from left ($x$~0) to right ($x$~1) for direct vertical comparisons of the side panels, i.e., (**b**) vs. (**c**) and (**f**) vs. (**e**), with (**d**) in-between offering a snapshot at intermediary composition. (**a**) Theoretical overview of the Cd_1−x_Be_x_Te TO Raman frequencies (curves) and intensities (color of curves) at 0 GPa within a four-mode $\left\{2\times \left(Cd-Te\right),2\times (Be-Te)\right\}$ description in absence of mechanical coupling (${\omega }{\prime}$=0). A sensitivity of bond vibrations to first neighbors is assumed, as for Zn_1−x_Be_x_Te^28^. The current experimental TO and LO Raman frequencies (hollow symbols) are indicated. BeTe data from the literature^48^ (filled symbols) are added for reference purpose. The prototypical parent-like supercell (216-atom) containing one isolated impurity-duo used to generate an ab initio insight into the end (*x* ~ 0,1) Cd_1−x_Be_x_Te TO Raman frequencies is sketched out. Labels (i) to (iv) refer to in-chain, out-of-chain, near-chain and away-from-chain bond vibrations, as indicated. The Be-Te doublet due to the sole effect of the local strain, i.e., in absence of dispersion, is schematically represented (straight-dashed lines). The dispersion effect affecting the impurity modes ($x$~0,1) is emphasized (vertical arrows). (**b**) High-pressure/low-temperature Cd_0.89_Be_0.11_Te Raman spectra in the upstroke. The Be–Te signal, modeled via Lorentzian functions (dotted lines) transiently exhibits a minor feature at ~ 3.5 GPa (asterisk). (**c,d,e**) ab initio (AIMPRO) pressure dependence of the Be–Te Raman signal **(c)** due to the Be-duo ($x\sim 0$), (**d**) at intermediary Be content ($x=0.5$) and (**e**) in presence of the Cd-duo ($x\sim 1$), giving rise to various local modes (asterisks). (**f**) Raman cross section of the irregular-inverted (see text) Be–Te TO Raman doublet depending on pressure at minor Cd content ($x\sim$ 0.81). A minor mechanical coupling is considered (${\omega }{\prime}$=50 cm^−1^). Straight and dotted lines represent the pressure dependencies of the raw-uncoupled Be-Te frequencies. In panels (**b**,**c**), a color code is used to distinguish between the raw-uncoupled TO’s stemming from “same” (red) and “alien” (blue) environments. Paired vertical—horizontal arrows in panels (**c**–**f**) emphasize pressure-induced changes in Raman intensity—frequency for a given mode, correspondingly.

Figure 2a displays an overview of the four-mode $\left\{2\times \left(Cd-Te\right), 2\times (Be-Te)\right\}$ TO Cd_1−x_Be_x_Te Raman frequencies (curves) and intensities (color code) in absence of mechanical coupling (see below) by analogy with Zn_1−x_Be_x_Te^28^. This was obtained by assimilating the Raman cross section with the imaginary part of the relative dielectric function ${\varepsilon }_{r}$, that captures the ${\varepsilon }_{r}\to \infty$ divergence characteristic of a purely-mechanical TO^47^ (Sect. SIII). Such overview offers a convenient eye-support for the forecoming discussion of the $x$-dependence of the Be–Te Raman doublet of Cd_1−x_Be_x_Te. Experimental TO and LO Raman frequencies of BeTe taken from the literature^48^ (filled symbols) are added, for reference purpose. The ab initio TO frequencies are slightly shifted upwards with respect to the current experimental values, by a few cm^-1^ for pure CdTe (hollow triangle in Fig. 2a) and by less than 15 cm^−1^ for pure BeTe (filled square). The shift is due to a generally known bias of the local density approximation, innate to our ab initio calculations, to overbind and hence to overestimate the bond force constants. An exception is the experimental Be–Te impurity mode ($x$~0, hollow circle) for which a nearly perfect matching occurs. The given overview unveils in advance a remarkable Be-Te feature, namely, an irregular crossing of the two Be–Te “percolation” sub-branches ($x$~0.8) contrasting with the regular parallelism of the Cd–Te ones.

Raman measurements at 0 GPa and 300 K with the red (632.8 nm) and blue (488.0 nm) laser lines, that are nearly resonant with the ${E}_{0}$ and ${E}_{0}+{\Delta }_{0}$ electronic transitions of Be-dilute Cd_1−x_Be_x_Te (Fig. 1b), respectively, fail to reveal the Be–Te doublet (Fig. S8). Only one Be–Te mode is visible, at ~ 390 cm^−1^, far away from the TO to LO CdTe-lattice band covering 140–170 cm^−1^. This is consistent with the scarce experimental far-infrared data in the literature^49^ and existing calculations using the Green’s function theory^50^. This line is assigned as the upper-main ${TO}_{Be-Te}^{Cd}$, by analogy with ${TO}_{Be-Te}^{Zn}$ of Zn_1−x_Be_x_Te. The lower/minor ${TO}_{Be-Te}^{Be}$ is not visible, presumably screened by the second-order CdTe-lattice signal, noted 2 ${LO}_{Cd-Te}$, emerging as a strong feature nearby. Basically, 2 ${LO}_{Cd-Te}$ is emphasized when using the red laser excitation, and reduced with the blue one.

The conditions for testing the PM are improved by keeping the blue laser line but working at high pressure and low temperature, i.e., 80 K. This offers a number of benefits. First, the Be–Te signal sharpens due to the increased phonon lifetime. Second, the pressure domain of the native zincblende phase enlarges^51^. Third, the low temperature slows down the formation of Te aggregates under intense laser exposition, that is a notorious problem with CdTe-like crystals^52^. The latter issue is especially critical when focusing the laser beam onto a tiny sample placed in a diamond anvil cell. The Raman spectra taken in the upstroke up to 4.3 GPa in the native zincblende phase of Cd_0.89_Be_0.11_Te (Fig. 2b) transiently reveal the target lower-minor ${TO}_{Be-Te}^{Be}$, marked by an asterisk, on the low-frequency side of the upper-main ${TO}_{Be-Te}^{Cd}$ at ~ 3.5 GPa, before its partial resorption at ~ 4.3 GPa. This is consistent with ${TO}_{Be-Te}^{Be}$ suffering a progressive collapse while converging towards ${TO}_{Be-Te}^{Cd}$ prior to its extinction at the crossing/resonance. The same phenomenon was observed with the reference Be-Te doublet of Zn_0.89_Be_0.11_Te across a similar pressure range (2.3–7.9 GPa)^28^. This fits into scenario 1 of the convergence process—cf. the issue 4.

Although the analogy with Zn_89_Be_11_Te is enlightening, it remains limited to grasp the behaviour of Cd_0.89_Be_0.11_Te. Additional support is searched for by calculating the high-pressure ab initio (AIMPRO) Cd_1−x_Be_x_Te Raman spectra at small-to-moderate Be content ($x$~0, 0.5). However, a limit to pressure is set by the supercells becoming unstable from 10 GPa ($x$~0, not shown) and 15 GPa ($x$~0.5, Fig. S7) onwards. The stability improves at large Be content ($x$~1), used to complete an ab initio Raman insight at well-spanned $x$ values across the composition domain (Fig. 2c,d,e).

Paired impurities forming a duo connected via Te in parent-like supercells, as schematized in Fig. 2a, represent the minimal impurity motif offering a clear distinction, in the context of the PM, between “impurity” and “host” vibrations in “same” and “alien” environments (labels $i$-to-$iv$ in Fig. 2a). This generates four situations in total at both ends of the composition domain ($x$ ~ 0 in Fig. 2c and $x$ ~ 1 in Fig. 2e). Detail is given in Sect. SII. Additional insight at maximum alloy disorder ($x=0.5$ in Fig. 2d) using a nominally random Cd_54_Be_54_Te_108_ supercell completes the ab initio picture.

Ab initio results reveal a Cd–Te percolation doublet for Cd_1−x_Be_x_Te at $x$~0 (Fig. S6a) and at $x$~1 (Fig. S6b), albeit a compact one (Fig. 2a)—cf. the issue 1, with ${TO}_{Cd-Te}^{Be}$ set below ${TO}_{Cd-Te}^{Cd}$, as expected—cf. the issue 2. The frequency gap ${\Delta }_{Cd-Te}$ hardly exceeds a few cm^−1^ that won’t be detectable in experiment. Hence, the Cd–Te vibration is almost blind to the local environment. At $x$~1 the upper ${TO}_{Cd-Te}^{Cd}$, spotted by an arrow in Fig. S6b, is frozen due to a phonon exceptional point being achieved already at 0 GPa—cf. the issue 4. In practice, the frozen mode is identified through its wavevector. At $x$~0 the Cd–Te doublet becomes inverted by increasing pressure to 5 GPa (Fig. S6a)—cf. the issues 3 and 4. Altogether, this offers a perfect analogy with the Zn-Te doublet of the reference Zn_1−x_Be_x_Te case^28^.

The analogy between Cd_1−x_Be_x_Te and Zn_1−x_Be_x_Te is not as clear in their common Be–Te spectral range, subsequently addressed with Cd_1−x_Be_x_Te at small, intermediary and large Be contents.

At $x$~0 (Fig. 2c), the Be-duo generates a nominal percolation-type (lower in-chain ${TO}_{Be-Te}^{Be}$, upper out-of-chain ${TO}_{Be-Te}^{Cd}$) Raman doublet at 0 GPa with a large ${\Delta }_{Be-Te}$ separation of $\sim 35$ cm^−1^—cf. the issue 2. Not surprisingly, the corresponding (${TO}_{Be-Te}^{Be},{TO}_{Be-Te}^{Zn}$) doublet in Zn_1−x_Be_x_Te is less resolved^28^, i.e., ${\Delta }_{Be-Te}\sim 20$ cm^-1^, due to the smaller bond ($\frac{\Delta l}{l},$ $\frac{\Delta {\alpha }_{c}^{3}}{{\alpha }_{c}^{3}}$)-contrast in Zn_1−x_Be_x_Te ($\sim 9\%,$ $\sim 31\%$) than in Cd_1−x_Be_x_Te ($\sim 15\%,$ $\sim 36\%$). By increasing pressure, ${TO}_{Be-Te}^{Be}$ gets closer to ${TO}_{Be-Te}^{Cd}$—cf. the issue 3, and suffers a major collapse, emphasized by paired arrows in Fig. 2c, to such extent that both its Raman intensity and ${\Delta }_{Be-Te}$ are halved. These are early signs of a phonon exceptional point on the verge of being achieved, in line with scenario 1—cf. the issue 4. Such trends find echo in experiment. However, ${TO}_{Be-Te}^{Be}$ shows up as a mere shoulder on ${TO}_{Be-Te}^{Cd}$ in experiment, marked by an asterisk in Fig. 2b, whereas it emerges as a distinct feature in ab initio data (Fig. 2c). This might relate to a basic difference at minor Be content that ab initio calculations run on an “ideal” lattice, verified by the ${B}_{0}$ versus $x$ linearity, whereas the real lattice suffers a massive ${B}_{0}$-drop in experiment (Fig. 1d).

At $x$~0.5 (Fig. 2d), scenario 1 still applies, as expected—cf. the issue 4. At 0 GPa, the Be–Te Raman signal of Cd_54_Be_54_Te_108_ ($x=0.5$) shows up as a unique broad ${TO}_{Be-Te}$ band. At 5 GPa, this transforms into a compact percolation-type (${TO}_{Be-Te}^{Be},{TO}_{Be-Te}^{Cd}$) doublet subsided on its low-frequency side. At 10 GPa, the doublet further shrinks and the subsidence is emphasized, as schematically indicated by paired arrows in Fig. 2d. Eventually, only ${TO}_{Be-Te}^{Cd}$ survives, and ${TO}_{Be-Te}^{Be}$ is killed. This nicely recapitulates the sequence leading to the achievement of a phonon exceptional point—cf. the issue 4. The sequence is interrupted from 15 GPa on, due to the collapse of the zincblende structure in the supercell used. This is manifested by a departure of bond angles from the nominal tetrahedral value of 109° (Fig. S7).

At $x$~1 (Fig. 2e), the situation at 0 GPa becomes irregular. While well separated when stemming from the Be-duo ($x$~0), the (${TO}_{Be-Te}^{Be},{TO}_{Be-Te}^{Cd}$) doublet of Cd_1−x_Be_x_Te becomes indiscernible when due to the Cd-duo ($x$~1). Indeed, minor features composing ${TO}_{Be-Te}^{Cd}$, marked by asterisks in Fig. 2e, do overlap with the main ${TO}_{Be-Te}^{Be}$. In contrast, the reference (${T}_{Be-Te}^{Be},{TO}_{Be-Te}^{Zn}$) doublet of Zn_1−x_Be_x_Te is globally preserved across the composition domain^28^.

The quasi degeneracy of the Be–Te Raman doublet of Cd_1−x_Be_x_Te at $x$~1 from 0 GPa undermines the possibility for scenario 2 to develop under pressure—cf. the issue 4, offering a novel case study.

Under pressure, the ${TO}_{Be-Te}^{Be}$ and ${TO}_{Be-Te}^{Cd}$ lines, already close at 0 GPa, are forced to further proximity, as indicated by a horizontal arrow in Fig. 2e, because the local environment becomes less discriminatory between like bond vibrations^28^. By getting closer, the two oscillators do couple mechanically. This mediates a transfer of oscillator strength from the main mode to the minor one, directly impacting the Raman intensities. Remarkably, the Be-Te oscillator strength is channeled from high to low frequency in the Cd_1−x_Be_x_Te case, as emphasized by the vertical arrow in Fig. 2e, and not the other way around as observed in the reference Zn_1−x_Be_x_Te ($x$~1) case^28^. This can be explained only if the minor ${TO}_{Be-Te}^{Cd}$, recipient of oscillator strength, lies below the main ${TO}_{Be-Te}^{Be}$, donor of oscillator strength, at 0 GPa. Hence, at 0 GPa the Be-Te doublet of Cd_1−x_Be_x_Te ($x$~1) is inverted relative to its regular Zn_1−x_Be_x_Te counterpart ($x$~1), and not only degenerated. Specifically, the vibration lines follow upwards in the (${TO}_{Be-Te}^{Cd}$, ${TO}_{Be-Te}^{Be}$) order, as opposed to the regular (${TO}_{Be-Te}^{Be}$, ${TO}_{Be-Te}^{Zn}$) order with Zn_1−x_Be_x_Te^28^ ($x$~1)—cf. the issue 2. The inversion is apparent in the superscript.

Generally, the shape of a given TO percolation doublet in a “Raman frequency vs. $x$” plot, as to whether the branches are parallel or suffer a trapezoidal or triangular distortion, manifests a compromise between the effects of the local strain and of the phonon dispersion^53^. The TO dispersion is markedly negative for BeTe^54^, i.e., of ~ 45 cm^-1^. As indicated by arrows at $x$~0,1 in Fig. 2a, this challenges the parallelism of the Be–Te doublet due to the sole effect of the local strain, represented by the dotted lines in Fig. 2a, leading to an inversion at $x$~1. More detail is given in Sect. SII in line with Ref.^53^. In contrast, the TO mode of CdTe is nearly dispersionless^55^. Hence, the parallelism is preserved for the Cd–Te doublet of Cd_1−x_Be_x_Te (Fig. 2a).

An interesting issue is how the mechanical coupling develops between the inverted Be–Te submodes when forced to proximity by pressure. The discussion is conducted hereafter in reference to the uncoupled case, modeled in Fig. 2a.

Fair modeling of the pressure-induced intensity interplay between the ab initio Raman submodes forming the irregular-inverted (${TO}_{Be-Te}^{Cd}$, ${TO}_{Be-Te}^{Be}$) percolation doublet of Cd_1−x_Be_x_Te near the crossing of percolation branches ($x$~1, Fig. 2e) is achieved within a dielectric parametrization of two (${TO}_{Be-Te}^{-}$, ${TO}_{Be-Te}^{+}$) mechanically-coupled harmonic oscillators (Fig. 2f). In doing so, we slightly adapt the approach developped in Ref.^28^, notably using there a simplified form of Eq. (7). detail is given in Sect. SIII. Hence, the inversion process manisfested in the high-pressure ab initio data (Fig. 2e) reflects the free mechanical coupling of Be–Te oscillators across the resonance—cf. the issue 4.

The free Be-Te coupling is also observed with Zn_1−x_Be_x_Te ($x$~1)^28^. Only, the coupling-induced transfer of Be-Te oscillator strength is opposite. This is because the Be-Te Raman doublets of Zn_1−x_Be_x_Te and Cd_1−x_Be_x_Te are inverted at 0 GPa, as discussed above. A further difference relates to the Be–Te convergence rate under pressure at $x$~1. This is fast for Zn_1−x_Be_x_Te^28^, with the inversion being completed already at ~ 10 GPa, and slow for Cd_1−x_Be_x_Te, the inversion being still in progress at the largest tested pressure of 20 GPa in our ab initio data (Fig. 2e). In the latter case, the inversion is even delayed to ~ 30 GPa in view of our dielectric parametrization of Raman lineshapes (Fig. 2f). Such discrepancy reflects different driving forces behind the pressure-induced Be-Te convergence processes. For Zn_1−x_Be_x_Te, the driving force is the regular $\partial {f}_{i}/\partial lnV$-mechanism—cf. the issue 3. For Cd_1−x_Be_x_Te, the convergence merely proceeds from a basic trend for like bonds to behave uniformly under pressure^28^. Anyway, the free coupling of the irregular-inverted Be-Te doublet of Cd_1−x_Be_x_Te at large Be content (Fig. 2e,f) opposes the phonon exceptional point achieved at minor-to-moderate Be content (Fig. 2b,c,d). This conforms to scenarii 2 and 1 in the main lines—cf. the issue 4.

## Conclusion

Among Be-based highly mismatched alloys (HMA’s), Cd_1−x_Be_x_Te achieves maximum contrast in bond properties, clarifying its vibrational-mechanical properties. As such, Cd_1−x_Be_x_Te is an appealing benchmark to test the limit of the percolation model (PM) that so far provided a unified understanding of the Raman spectra of semiconductor alloys, covering well matched alloys (WMA’s) and HMA’s.

${B}_{0}$ suffers a counterintuitive drop below the CdTe value on minor Be incorporation ($x$~0), a sign of degraded mechanical properties at the macroscopic scale. At the microscopic scale the Be-Te PM-type Raman doublet at large Be content ($x$~1) is fully relaxed by the phonon dispersion, down to inversion, an unprecedented case. The PM is not challenged for all that and basically applies as such to Cd_1-x_Be_x_Te. In fact, Be–Te exhibits a mere bimodal Raman pattern at any composition, and not a more complicated one. Further, the pressure-induced convergence of the Be–Te Raman doublet either ends up into a phonon exceptional point ($x$~0–0.5) or develops into a free mechanical coupling ($x$~1) depending on the bond fraction, in line with PM-predictions. This reinforces the status of the PM as a robust descriptor of phonons in semiconductor alloys.

## Methods

This section provides details on the samples and various experimental techniques and simulation (numerical and analytical) methods used to probe and to support the discussion on the structural, electronical and vibrational properties of Cd_1−x_Be_x_Te, depending on pressure.

### Samples

Cd_1−x_Be_x_Te ($x=0$, 0.03, 0.05, 0.07, 0.11) and Zn_1−x_Be_x_Te ($x=0.04$, 0.21) free-standing single crystals required for high-pressure Raman and X-ray diffraction measurements are grown from the melt by mixing high-purity CdTe and ZnTe (99.9995, i.e., 5 N quality) with Be (99.5, i.e., 2 N quality) using the Bridgman method^56^. The Cd_1−x_Be_x_Te composition is determined better than 1% by selective Cd and Be dosing via the inductively coupled plasma (ICP) method applied to powders. All samples crystallize in the native zincblende structure of CdTe and BeTe, verified by powder X-ray diffraction measurements at 0 GPa (Sect. SI). The Zn_1−x_Be_x_Te composition is derived from the linear composition dependence of the lattice constant^34^ measured at 0 GPa by powder X-ray diffraction.

### Solid-state nuclear magnetic resonance measurements

Solid-state nuclear magnetic resonance (SS-NMR) measurements of the chemical shift due to the invariant Te species of Cd_1-x_Be_x_Te are performed on a finely ground powder (~ 20 ml) obtained from the largest crystal piece at hand ($x$= 0.07). The SS-NMR insight is needed to test whether the Cd $\leftrightarrow$ Be atom substitution is ideally random, or not, *i.e.*, subject to clustering or anticlustering. For all solid-state NMR spectra, Bruker 2.5 mm double resonance probes are used and the magic angle spinning frequency is set to 25 kHz. ^125^Te, ^113^Cd and ^9^Be longitudinal relaxation times (${T}_{1}$), respectively 450, 840 and 175 s, are measured using standard saturation recovery experiments. Recycle delays of 5 times ${T}_{1}$ are then used to ensure quantitativity. ^125^Te NMR spectrum is acquired on a Bruker Avance III spectrometer operating at 7 T corresponding to 300 MHz ^1^H resonance frequency. The direct-acquisition Carr-Purcell-Meiboom-Gil (CPMG) experiment^57^ enables to record, in each scan, 49 full echoes with 400 ms acquisition time. 256 scans are accumulated in 7 days and 9 h. ^113^Cd and ^9^Be NMR standard direct acquisition experiments are recorded on a Bruker Avance III 600 MHz spectrometer (14 T). Respectively 112 and 56 transients are recorded, for a total experimental time of 5 days 10 h for ^113^Cd and 15 h for ^9^Be.

### Ellipsometry measurements

Near the direct absorption edge, the square root of the deviation ($E-{E}_{0}$) from the fundamental band gap (${E}_{0}$) scales linearly with the energy ($E$) weighted by the absorption coefficient ($\alpha$). ${E}_{0}$ is obtained by measuring such so-called Tauc plots^58^ in the visible using non-oriented crystal pieces with parallel faces polished to optical quality. The measurements are done both directly, in a transmission experiment at normal incidence, and indirectly, by ellipsometry at a near-Brewster incidence. In the latter case a HORIBA UVISEL phase modulated spectroscopic ellipsometer is used. Higher interband transitions (${E}_{1}$, ${E}_{1}+{\Delta }_{1}$, ${E}_{2}$) on top of ${E}_{0}$ are accessed via a direct, i.e., model-free, wavelength-per-wavelength inversion of spectrometric ellipsometry data, namely the sine and cosine of the depolarization angles.

### (High-pressure) X-ray diffraction measurements

High-pressure powder X-ray diffractograms are recorded on the PSICHÉ (Cd_0.89_Be_0.11_Te) and CRISTAL (Zn_1−x_Be_x_Te, $x=0.045$, 0.14 and 0.21) beamlines of SOLEIL synchrotron using radiation wavelengths of 0.3738 and 0.485 Å, respectively. The high-pressure data are recorded with a similar Chervin type diamond anvil cell^59^ as used for the Raman measurements, with 300 μm in diameter diamond culet. In both PSICHÉ and CRISTAL measurements, Ne and Au are used as the pressure transmitting medium and for pressure calibration, respectively. The high-pressure X-ray data are treated by using the DIOPTAS^60^ software at PSICHÉ and the DATLAB software at CRISTAL. The latter was kindly made available on site by K. Syassen (Max-Planck Institut für Festkörperphysik, Stuttgart, Germany). The ${B}_{0}$ value of the native zincblende phase at 0 GPa is obtained by fitting the Birch–Murnaghan equation of state to the pressure dependence of the unit cell volume^41^. The volume of the unit cell at 0 GPa coming into the cited equation is determined from powder X-ray diffraction measurement done at 0 GPa in laboratory conditions using the CuKα radiation.

### (High-pressure) Raman scattering measurements

High-pressure and low-temperature Raman experiment on Cd_0.89_Be_0.11_Te is done in the backscattering geometry. The laser beam is focused through a $\times$ 50 microscope objective with a long working distance at normal incidence onto a 40 µm in diameter spot at the non-oriented surface, polished to optical quality, of a tiny monocrystal inserted into a Chervin type diamond anvil cell (described above). The cell is set into a Helium flow cryostat system operated at liquid nitrogen temperature. CdTe-based crystals being notoriously poor Raman scatterers^52^, near-resonant conditions are used to enhance the Raman signal. Such conditions are best achieved at 300 and 77 K by using the 632.8 nm line of a helium–neon laser, falling in between ${E}_{0}$ and ${E}_{0}+{\Delta }_{0}$, and the 488.0 nm line of an argon laser, near-resonant with ${E}_{0}+{\Delta }_{0}$, respectively. Fair modeling of the Raman signal due to uncoupled or mechanically-coupled TO oscillators is achieved within a linear dielectric function approach, adapted from Ref.^28^ (Sect. SIII).

### (High-pressure) ab initio insights into the lattice relaxation/dynamics

Three ab initio codes operated within the density functional theory, using pseudopotentials and the local density approximation for the exchange–correlation, are employed, according to need. Reference ${B}_{0}$ versus $x$ curves for Cd_1−x_Be_x_Te at moderate Be content ($x\le 0.3$) are independently obtained by applying the SIESTA^32,33^ and AIMPRO (Ab Initio Modeling PROgram^29,30^) codes to 64- and 216-atom fully-relaxed and partially-relaxed disordered zincblende-type supercells, respectively. The full relaxation is concerned with the lattice constant, the atom positions and the supercell shape. The partial relaxation is done so as to prevent any supercell distortion and hence maintain the cubic structure needed for a strict application of the Birch–Murnaghan^41^ equation of state. Each supercell represents a random Cd $\leftrightarrow$ Be substitution, obtained by adjusting the fractions of individual Te-centred tetrahedra to the binomial Bernoulli distribution^36^. At each $x$ value, ${B}_{0}$ is estimated by fitting the pressure dependence of the volume unit cell with the Birch-Murnaghan equation of state^41^. Further AIMPRO Raman calculations, using the formula given by de Gironcoli^61^, are done on parent-like supercells containing Be-($x$~0) or Cd-paired ($x$~1) impurity motifs. The motivation is to imitate the limiting-cases Raman signals due to the non-polar, i.e., purely-mechanical, TO’s of the host and impurity species. In their current versions the AIMPRO and SIESTA codes do not take into account the long-range electric field accompanying the Raman active polar LO’s. Further simulations thus resorted to QE code^33^, giving access to the frequency ${\omega }_{T}$ of the non-polar TO, to the frequency ${\omega }_{L}$ of the Raman-active polar LO and to the high-frequency relative dielectric constant ${\varepsilon }_{\infty }$, defined at $\omega \gg$ ${\omega }_{T}$, of each parent compound. The QE calculations are performed on 2 $\times$ 2 $\times$ 2 cubic zincblende-type 64-atom supercells depending on pressure. From these, the static relative dielectric constant ${\varepsilon }_{s}$, defined at $\omega \ll$ ${\omega }_{T}$, could have been extracted by force of the LST relation^62^, giving access to the parent phonon oscillator strength, i.e., ${\varepsilon }_{s}-{\varepsilon }_{\infty }$. This is a major ingredient in the classical form of the relative Cd_1−x_Be_x_Te dielectric function ${\varepsilon }_{r}$, that monitors the resonant term in our simplified analytical expression of the Cd_1−x_Be_x_Te Raman cross section (Sect. SIII).

### Supplementary Information

Supplementary Information.

## Data Availability

The reported experimental (high-pressure Raman, high-pressure X-ray diffraction, nuclear magnetic resonance) and ab initio (AIMPRO, SIESTA and QE codes) data in this work are available upon request to the corresponding author.

## References

[1] Powalla M (2018). Thin-film solar cells exceeding 22% solar cell efficiency: An overview on CdTe-, Cu(In, Ga)Se2-, and perovskite-based materials. Appl. Phys. Rev..

[2] Adachi S (2009). Properties of semiconductor alloys: Group-IV, III–V and II–VI.

[3] Christensen NE, Satpathy S, Pawlowska Z (1987). Bonding and ionicity in semiconductors. Phys. Rev. B.

[4] Vérié C (1998). Beryllium substitution-mediated covalency engineering of II-VI alloys for lattice elastic rigidity reinforcement. J. Cryst. Growth.

[5] Weyers M, Sato M, Ando H (1992). Red shift of photoluminescence and absorption in dilute GaAsN alloy layers. Jpn. J. Appl. Phys..

[6] Lugauer H-J (1997). P-type doping of beryllium chacogenides. Mater. Sci. Eng. B.

[7] Waag A (1998). Novel Beryllum containing II-VI compounds: Basic properties and potential applications. J. Cryst. Growth.

[8] Ahrafi AA (2000). Growth and characterization of hypothetical zincblende ZnO films on GaAs (001) substrates with ZnS buffer layers. Appl. Phys. Lett..

[9] Walukiewicz W, Zide JMO (2020). Highly mismatched semiconductor alloys: From atoms to devices. J. Appl. Phys..

[10] Neugebauer J, Van de Walle CG (1995). Electronic structure and phase stability of GaAs_1-x_N_x_ alloys. Phys. Rev. B.

[11] Wei S-H, Zunger A (1996). Giant and composition-dependent optical bowing coefficient in GaAsN alloys. Phys. Rev. Lett..

[12] Hjalmarson H (1980). Theory of substitutional deep traps in covalent semiconductors. Phys. Rev. Lett..

[13] Shan W (1999). Band anticrossing in GaAsN alloys. Phys. Rev. Lett..

[14] Wu J, Walukiewicz W, Haller EE (2002). Band structure of highly mismatched semiconductor alloys: Coherent potential approximation. Phys. Rev. B.

[15] Chen C (2019). Carrier dynamics of intermediate sub-bandgap transitions in ZnTeO. J. Appl. Phys..

[16] Tanaka T (2019). Cl-doping effect in ZnTe_1__−__x_O_x_ highly mismatched alloys for intermediary band solar cells. J. Appl. Phys..

[17] Allami, H. & Krich, J. J. Absorption spectrum of doped highly mismatched alloys. arXiv:2203.15528 [cond-mat.mtrl-sci].

[18] Maksimov O, Tamargo MC (2001). Direct-to-indirect band gap crossover for the Be_x_Zn_1-x_Te alloy. Appl. Phys. Lett..

[19] Chauvet C, Tournié E, Faurie J-P (2000). Nature of the band gap of Zn_1-x_Be_x_Se alloys. Phys. Rev. B.

[20] Wilmers K (1999). VUV-Ellipsometry on Be_x_Zn_1-x_Se and BeTe. J. Electron. Mater..

[21] Buckley MR (2002). Dielectric functions and critical points of Be_x_Zn_1-x_Te alloys measured by spectroscopic ellipsometry. Appl. Phys. Lett..

[22] Wilmers K (1999). Ellipsometric studies of Be_x_Zn_1-x_Se between 3 eV and 25 eV. Phys. Rev. B.

[23] Kozielski M (1999). Study if the A_1-x_B_x_C mixed crystals by Raman scattering. Cryst. Res. Technol..

[24] Mintairov AM (1999). Optical spectra of wide band gap Be_x_Zn_1-x_Se alloys. Semiconductors.

[25] Pagès O (2001). Vibrational evidence for a percolative behavior in Zn_1-x_Be_x_Se. Phys. Rev. B.

[26] Chang IF, Mitra SS (1971). Long wavelength optical phonons in mixed crystals. Adv. Phys..

[27] Shoker, M. B. *et al*. Phonon-based partition of (ZnSe-like) semiconductor mixed crystals on approach to their pressure-induced structural transition. *Sci. Rep.* **10,** 19803 (2020)—and refs. therein. 10.1038/s41598-020-76509-0 PMC7666148 33188245

[28] Shoker MB (2022). Exceptional phonon point versus free phonon coupling in Zn_1-x_Be_x_Te under pressure: An experimental and ab initio Raman study. Sci. Rep..

[29] Briddon PR, Jones R (2000). LDA calculations using a basis of Gaussian orbitals. Phys. Stat. Sol. B.

[30] Rayson MJ, Briddon PR (2008). Rapid iterative method for electronic-structure eigenproblems using localized basis functions. Comput. Phys. Commun..

[31] Soler JM (2002). The SIESTA method for ab-initio order-N materials simulation. J. Phys. Condens. Matter.

[32] García A (2020). SIESTA: Recent developments and applications. J. Chem. Phys..

[33] Giannozzi P (2009). Quantum Espresso: A modular and open-source software project for quantum simulations of materials. J. Phys. Condens. Matter.

[34] De Almeida JS, Ahuja R (2006). Tuning the structural, electronic and optical properties of Be_x_Zn_1-x_Te alloys. Appl. Phys. Lett..

[35] Wagner V (2003). Surface and overgrowth analysis of the II-VI compound BeTe. Appl. Surf. Sci..

[36] Zamir D (1988). Nuclear magnetic resonance studies of II-VI semiconductor alloys. J. Vac. Sci. Technol. A.

[37] Cardona M, Greenaway DL (1963). Fundamental reflectivity and band structure of ZnTe, CdTe and HgTe. Phys. Rev..

[38] Hododyský P, Hlídek P (2006). Free-exciton absorption in bulk CdTe: Temperature dependence. Phys. Stat. Sol. (b).

[39] Mujica A, Rubio A, Muñoz A, Needs RJ (2003). High-pressure phases of group-IV, III-V and II-VI compounds. Rev. Mod. Phys..

[40] Bhalerao GM (2010). High-pressure X-ray diffraction and extended X-ray absorption fine structure studies on ternary alloy Zn_1-x_Be_x_Se. J. Appl. Phys..

[41] Birch F (1947). Finite elastic strain of cubic crystals. Phys. Rev..

[42] McMahon MI, Nelmes RJ, Wright NG, Allan DR (1993). Phase transitions in CdTe to 5 GPa. Phys. Rev. B.

[43] Luo H, Ghandehari K, Greene RG, Ruoff AL (1995). Phase transformations of BeSe and BeTe to the NiAs structure at high pressure. Phys. Rev. B.

[44] Pellicer-Pores J (2005). High-pressure phase diagram of ZnSe_1-x_Te_x_ alloys. Phys. Rev. B.

[45] Zunger A, Wei S-H, Ferreira LG, Bernard JE (1990). Special quasirandom structures. Phys. Rev. Lett..

[46] Wei S-H, Ferreira LG, Bernard JE, Zunger A (1990). Electronic properties of random alloys: Special quasirandom structures. Phys. Rev. B.

[47] Claus, R., Merten, L. & Brandmüller, J. in *Light scattering by Phonon-Polaritons*, (ed Höhler, G.) (Springer, 1975), p. 62.

[48] Pagès O (2002). Percolation behavior in the Raman spectra of ZnBeTe alloys. Appl. Phys. Lett..

[49] Sennet CT, Bosomworth DR, Hayes W, Spray AR (1969). Infra-red absorption of beryllium in cadmium telluride: II. J. Phys. C.

[50] Talwar DN, Feng ZC, Yang T-Z (2012). Vibrational signatures of isotopic impurities and complexes in II-VI compound semiconductors. Phys. Rev. B.

[51] Gilliland S (2003). Pressure and temperature dependence of the band-gap in CdTe. Phys. Stat. Sol. (b).

[52] Talwar DN, Feng ZC, Lee J-F, Becla P (2014). Extended X-ray absorption fine structure and micro-Raman spectra of Bridgman grown Cd_1-x_Zn_x_Te ternary alloys. Mater. Res. Express.

[53] Hajj Hussein R (2015). Percolation-type multi-phonon pattern of Zn(Se, S): Backward/forward Raman scattering and ab initio calculations. J. Alloys Compd..

[54] Srivastava GP, Tütüncü HM, Günhan N (2004). First-principles studies of structural, electronic and dynamical properties of Be chalcogenides. Phys. Rev. B.

[55] Rajput BD, Browne DA (1996). Lattice dynamics of II-VI materials using the adiabatic bond-charge model. Phys. Rev. B.

[56] Gorgol M (2021). Positron lifetime spectroscopy of defect structures in Cd_1-x_Zn_x_Te mixed crystals grown by vertical Bridgman-Stockbarger method. Acta Cryst. B.

[57] Larsen FH, Jakobsen HJ, Ellis PD, Nielsen NC (1997). Sensitivity-enhanced quadrupolar-echo NMR of half-integer quadrupolar nuclei. Magnitudes and relative orientation of chemical shielding and quadrupolar coupling tensors. J. Phys. Chem. A.

[58] Ganjoo A, Golovchack R (2008). Computer program PARAV for calculating optical constants of thin films and bulk materials/case study of amorphous semiconductors. J. Opt. Adv. Mater..

[59] Chervin JC, Canny B, Besson JM, Pruzan P (1995). A diamond anvil cell for IR microspectroscopy. Rev. Sci. Instrum..

[60] Prescher C, Prakapenka VB (2015). DIOPTAS: A program for reduction of two-dimensional X-ray diffraction data and data exploration. High Press. Res..

[61] De Gironcoli S (1992). Phonons in Si-Ge systems: An ab initio interatomic-force-constant approach. Phys. Rev. B.

[62] Klingshirn CF (1997). Semiconductor optics.

