# Supplementary material for: Vibrational-mechanical properties of the highly-mismatched Cd1−xBexTe semiconductor alloy: experiment and ab initio calculations
Source: Sci Rep. 2023 Sep 4;13:14571. doi: 10.1038/s41598-023-39248-6 (PMC10477277; doi:10.1038/s41598-023-39248-6)
Supplement: Supplementary file 1 — Supplementary Information. [file 41598_2023_39248_MOESM1_ESM.pdf]

Supplementary Information

Vibrational-mechanical properties of the highly-mismatched $Cd_{1-x}Be_xTe$ semiconductor alloy – Experiment and *ab initio* calculations

A. Elmahjoubi, M. B. Shoker, O. Pagès, V. J. B. Torres, A. Polian, A. V. Postnikov, C. Bellin, K. Béneut, C. Gardiennet, G. Kervern, A. En Naciri, L. Broch, R. Hajj Hussein, J.-P. Itié, L. Nataf, S. Ravy, P. Franchetti, S. Diliberto S. Michel, A. Abouais and K. Strzałkowski

In this annex, supplementary experimental, *ab initio* (AIMPRO) and modeling (linear dielectric approach) material is presented further supporting the discussion of the structural, optical (**Sec. SI**) and mechanical-vibrational properties of $Cd_{1-x}Be_xTe$ (**Sec. SII**) done in the main text. Raw experimental data concerned with the structural properties of Be-dilute $Cd_{1-x}Be_xTe$ studied at the lattice-macroscopic and bond-microscopic scales by high-pressure X-ray diffraction (HP-XRD) and by solid state nuclear magnetic resonance (SS-NMR), completing Fig. 1, are reported in **Secs. SI.1** and **SI.2**, correspondingly. In the former case a fruitful comparison with $Zn_{1-x}Be_xTe$ is included (HP-XRD, **Sec. SI.1**). **Sec. SI.3** reports on the main $Cd_{1-x}Be_xTe$ electronic interband transitions (E_0,E_1,$E_1+\Delta_1$), determined by combining transmission and ellipsometry measurements in the visible. **Sec. SII.** reports on the $Cd_{1-x}Be_xTe$ lattice relaxation and lattice dynamics *ab initio* calculations depending on pressure in the Be- (x~0) and Cd-dilute (x~1) limits and at intermediary composition (x=0.5). **Sec. II** is introduced by general considerations in **Sec. II.1** on the shape of the percolation-type Be-Te Raman doublet of $Cd_{1-x}Be_xTe$ in a "frequency vs. x" plot at ambient pressure, as the result of a competition between the effects of the local strain and of the phonon dispersion, as discussed in Ref.[53]. In **Sec. II.2**, an isolated Be impurity in CdTe, suffering a hydrostatic tensile strain from the host matrix, offers a benchmark to estimate in a full *ab initio* (SIESTA) approach how the local strain and the phonon dispersion separately impact the Raman frequencies of impurity modes. **Sec. II.3** completes the high-pressure *ab initio* Raman study of paired-impurities in parent-like supercell done in the main text (Figs. 2c and 2e) by shifting the focus to the Cd-Te spectral range. **Sec. II.4** provides an *ab initio* insight into the pressure dependence of the lattice relaxation within the $Cd_{54}Be_{54}Te_{108}$ cubic supercell used to calculate the *ab initio* high-pressure Raman spectra reported in Fig. 2d. **Sec. SIII** is concerned with the $Cd_{1-x}Be_xTe$ lattice dynamics. **Sec. III.1** reports on an experimental Raman study of the $Cd_{1-x}Be_xTe$ vibrational properties at minor Be contents done at 0 GPa in preparation for the high-pressure Raman study shown in Fig. 2b. Last, in **Sec. SIII.2** we outline the linear dielectric approach used for contour modeling of the various Raman lineshapes displayed in Fig. 2. We refer to the four-mode $\{TO_{Cd-Te}^{Be}, TO_{Cd-Te}^{Cd}, TO_{Be-Te}^{Be}, TO_{Be-Te}^{Cd}\}$ overview given across the composition domain at 0 GPa (**Sec. SIII.2.a**) in absence of mechanical coupling (ω'=0 cm^{-1}, Fig. 2a), and also to the punctual insight into the pressure dependence of the Be-Te Raman doublet at x~1 (Fig. 2f). This is achieved by considering a weak mechanical coupling, *i.e.*, ω'=50 cm^{-1}, impacting both the Raman frequencies and the Raman intensities (**Sec. SIII.2.b**).

I. Structural and optical properties of Be-dilute $Cd_{1-x}Be_xTe$

I.1. (High-pressure) X-ray diffraction – Structural insight at the macroscopic (lattice) scale

Raw X-Ray diffractograms obtained at 0 GPa in laboratory using the CuKα radation across the current $Cd_{1-x}Be_xTe$ (x ≤0.11) sample series (Fig. S1a) are characterized by sharp peaks at any composition x, the sign of a high structural quality. The individual peaks are labelled using the (hkl) Miller indices up

to maximal angular deviation. The lattice constant a is found to vary linearly with x (Fig. S1b), a standard feature of semiconductor alloys[2].

A selection of raw high-pressure X-ray diffractograms taken at increasing pressure on $Cd_{0.89}Be_{0.11}Te$ at the PSICHÉ beamline of SOLEIL synchrotron using the 0.3738 Å radiation (Fig. S1c) reveals no extra peak besides the regular ones for a given crystal phase, the sign of a high structural purity. As pressure increases, $Cd_{0.89}Be_{0.11}Te$ transforms from zincblende (0 GPa, abbreviated zb) to rocksalt (~5.5 GPa, rs), the two phases coexisting over a rather large pressure domain, *i.e.*, ~5.5 – 9 GPa. The crystal subsequently adopts the Cmcm structure at ~13 GPa, eventually surviving as a unique phase from ~18 GPa up to the maximum achieved pressure of ~24 GPa. The pressure dependence of lattice constants in each $Cd_{0.89}Be_{0.11}Te$ phase (Fig. 1c) is notably used to estimate the bulk modulus B_0 at 0 GPa in the native zincblende phase (Fig. 1d). This is done by fitting the pressure dependence of the unit cell volume to the Birch-Murnaghan equation of state[41] (Fig. S1d).

Fig. S2 displays selections of similar high-pressure X-ray diffractograms obtained with $Zn_{1-x}Be_xTe$ at (a) x=0.045 and (b) 0.21 at the CRISTAL beamline of SOLEIL synchrotron using the 0.485 Å radiation. The current data complete the earlier published x=0.14 data set[28] taken during the same run of experiment. The related experimental (symbols) volume versus pressure dependencies in the native zincblende phase fitted to the Birch-Murnaghan equation of state[41] (curves) and resulting B_0 vs. x variations are displayed in Figs. S3a and S3b, respectively. The fit is done by fixing the reference volume at 0 GPa to the value obtained by considering a linear dependence of the lattice constant versus x in $Zn_{1-x}Be_xTe$[34]. The as-fitted B_0 (symbols) and B_0' values at x=(0.045, 0.14, 0.21) are (52.857±0.640, 52.846±0.170, 52.542±0.150) in GPa and (3.94±0.18, 4.05±0.09, 4.03±0.06), respectively. The error bars are within the symbol size. For all mixed crystals, B_0' remains stable around 4, found relevant for ZnTe[44] and BeTe[43]. A pronounced deviation from the B_0 vs. x linearity (dashed line in Fig. S3b) in the sense of a negative bowing is observed, as in the case of $Cd_{1-x}Be_xTe$ (Fig. 1d). The minimal B_0 value is achieved at 21 at.% Be, quasi matched with the parent ZnTe value[44], *i.e.*, 52±1 GPa, well below the BeTe one[43], *i.e.*, 67±1 GPa.

I.2. Solid state nuclear magnetic resonance (SS-NMR) – Structural insight at the microscopic (atom) scale

Generally, the bond force constant k and the bond length l are related via a basic rule that the former decreases as the latter is stretched, and vice versa. Referring this to the $Zn_{1-x}Be_x$-HMA's, the variation in Be-related bond force constant of ~10% observed in Raman[25,27] and far-infrared absorption[S1] experiments is correlated with a predicted variation in Be-related bond length of ~2%, from *ab initio* calculations[S2], depending on whether the Be-related bond is in a Be- or Zn-like environment. Unfortunately, this bimodal distribution of the Be-related bond length could not be resolved in extended X-ray absorption fine structure (EXAFS) measurements on a synchrotron[S3,S4]. *A priori* such variation in bond length is also hardly detectable via the pair distribution function in conventional total X-ray scattering experiments[S5,S6]. The same applies a fortiori to the less contrasted WMA's. For instance, the covalent (III-V) $Ga_{1-x}In_xAs$ and ionic (II-VI) $ZnSe_{1-x}Te_x$ alloys usually ranked among WMA's despite their significant lattice mismatch[3], *i.e.*, ~7%, exhibit a unique bond length per species at a given x value in EXAFS data[S7-S9]. Yet their Raman signal is bimodal per bond, of the percolation type[25,S10]. Hence, EXAFS yields a basic 1-bond→1-length description[S11] whereas the Raman signal diversifies into a 1-bond→2-mode percolation-type pattern. Somehow, this suggests that the lattice dynamics zooms deeper into the alloy disorder than the lattice relaxation. This is interesting on the fundamental side.

The phonons being sensitive to the local environment, as formalized within the PM, they can be used to elucidate whether the atom substitution is ideally random or not[27], the central issue when dealing with alloys. Alternatively, one may resort to EXAFS measurements on a synchrotron of the second-neigbor distances for the common-atom sublattice, *i.e.*, the Te-Cd-Te and Te-Be-Te ones for $Cd_{1-x}Be_xTe$. These differ on account that each bond tends to preserve its natural length in an alloy[S8,S11]. Apart from vibrational spectroscopies, the solid-state nuclear magnetic resonance (SS-NMR) seems to

be the only one in-house technique capable of addressing such issue quantitatively[36], as exemplified below with $Cd_{1-x}Be_xTe$.

In an alloy with zincblende structure such as $Cd_{-x}Be_xTe$, the substituent (Cd and Be in this case) and invariant (Te) species are intercalated so a to form a cubic tetrahedral arrangement. Hence, Cd and Be exhibit a stable nearest-neighbor environment of four Te atoms at any x value. In contrast the tetrahedral environment of Te diversifies into five variants depending on the number of Be and Cd atoms at the vertices. The five types of Te-centrered tetrahedra are present in the crystal at any x value, with various probabilities depending on x, following the binomial Bernoulli's distribution in their x-dependence in the ideal case of a random Be↔Cd substitution[S12].

The pioneering ^{125}Te SS-NMR measurements performed on $Cd_{1-x}Zn_xTe$ by Zamir *et al.*[36] have demonstrated a sensitivity of the SS-NMR shift to the local environment at the nearest-neighbor scale. Hence, only the SS-NMR data related to the invariant Te species of $Cd_{1-x}Be_xTe$, as opposed to the substitutional Cd and Be ones, can shed light on the nature of the Cd↔Be atom substitution. This why the emphasis is put on Te in the main text (Fig. 1a). However, the ^{9}Be (a) and ^{113}Cd (b) NMR data related to both substituents are also provided (Fig. S4), for the sake of completeness. A unique well-defined feature, reflecting the uniqueness of the local environment (all-Te), is visible in each case, as ideally expected.

I.3. Optical properties – Transmission and ellipsometry in combination

The main $Cd_{1-x}Be_xTe$ electronic interband transitions are determined across the sample series in their x-dependence by combining transmission (E_0) and ellipsometry ($E_1, E_1 + \Delta_1, E_2$) measurements in the visible (Fig. 1b). Selected data (Fig. S5) illustrate how the interband transitions are estimated in practice from the raw spectrometric data at each composition. With the transmission data this is done via a Tauc plot (curve in Fig. S5a; filled symbols in Fig. 1b). With the ellipsometry data (Fig. S5b), the transitions (hollow symbols in Fig. 1b) are identified via a direct, *i.e.*, model-free, inversion of the measured depolarization angles. This gives access to the imaginary part of the dielectric function within the 0.6 – 5.5 eV spectral range.

II. $Cd_{1-x}Be_xTe$ lattice relaxation / dynamics – *Ab initio* insights

II.1. $Cd_{1-x}Be_xTe$ "TO Raman frequency vs. x" plot: shape of the Be-Te Raman doublet.

In earlier work[53] we have shown that the shape of the upper, and best-resolved, TO percolation doublet in a "Raman frequency vs. x" plot may vary a lot, as to whether the branches are ideally parallel or suffer a trapezoidal or triangular distortion. This depends on wether the parent TO mode is dispersive or not. The main arguments are briefly recalled hereafter by focusing on the lower parent$-iv$ and upper impurity$-iii$ modes forming the percolation doublet in the parent limit. Only the latter mode is subject to dispersion, not the former one[53]. Hence their comparison provides a straightforward insight into the dispersion effect.

In absence of dispersion, as, *e.g.*, for GaP, the $iii - iv$ frequency gap is solely governed by the local strain due to the bond mismatch, between, *e.g.*, Ga-As and Ga-P in the case of $GaAs_{1-x}P_x$. This results in parallel branches across the composition domain[53]. The TO mode of CdTe is nearly dispersionless[55], so that the Cd-Te doublet of $Cd_{1-x}Be_xTe$ actually exhibits such parallelism (Fig. 2a). In case of a positive (resp. negative) dispersion, iii is upward (resp. downward) shifted with respect to iv, roughly by the magnitude of the dispersion. The iii–iv frequency gap is accordingly enlarged (resp. squeezed), leading to a trapezoidal (resp. triangular) distortion of the doublet, as observed[53] with $ZnSe_{1-x}S_x$ (resp. $Si_{1-x}Ge_x$).

We are mainly interested in the negative TO dispersion. In this case an inversion of the percolation doublet occurs if the dispersion effect outweights that of the local strain, as observed, *e.g.*, with the Si-Si doublet[51] of $Si_{1-x}Ge_x$ at x~0. Such an inversion does not occur in the dilute limit, because, as impurity modes, the i and ii forming the percolation doublet therein suffer a similar dispersion effect. Since the percolation doublet is inverted in the parent limit but regular in the dilute one, its two sub-

branches should cross at a certain composition. Such crossing was not observed yet, not even with $Si_{1-x}Ge_x$, due to the i-ii degeneracy[53] at $x\sim1$.

The TO mode of BeTe exhibits a large negative dispersion[53], *i.e.*, ~50 cm^{-1}. Accordingly, in both $Zn_{1-x}Be_xTe$[28] and $Cd_{1-x}Be_xTe$ the Be-Te splitting is smaller in the parent limit ($x\sim1$) than in the dilute one ($x\sim0$). The exact spacings at $x\sim1$ are 7 cm^{-1} (Ref.[28]) and ~0 cm^{-1} (Fig. 2d), against ~30 cm^{-1} (Ref.[53]) and ~45 cm^{-1} (Fig. 2c) at $x\sim0$, respectively. Based on *ab initio* (SIESTA) calculations done in the next sub-section (SII.2) on the Be-Te impurity$-i$ mode of $Cd_{1-x}Be_xTe$, taken as representative for all i-to-iii Be-Te impurity modes, the Be-Te doublet at $x\sim0$, *i.e.*, i-ii, is downshifted as a whole by ~11 cm^{-1} due to the dispersion effect. This is emphasized by short vertical arrows in Fig. 2a at $x\sim0$. A crude view of the virtual Be-Te doublet of $Cd_{1-x}Be_xTe$ due to the sole effect of the local strain, *i.e.*, deprived of dispersion, is artificially reconstructed by drawing parallel Be-Te sub-branches (dashed lines), taken straight in a first approximation, between the virtual Be-Te doublet inferred at $x\sim0$ in absence of dispersion and the same doublet attached to the parent TO mode at $x\sim1$. In doing so, we proceed by analogy with the dispersionless Ga-P doublet of $GaAs_{1-x}P_x$[53].

When confronted with *ab initio* data at $x\sim1$, taking into account blindly the effects of strain and dispersion, the above-derived virtual Be-Te doublet dictated by the sole strain effect appears to be seriously challenged by the TO dispersion, whether considering $Cd_{1-x}Be_xTe$ or $Zn_{1-x}Be_xTe$[28]. In the latter case, however, the effect of the local strain dominates. Hence the ordering of Be-Te branches is nicely preserved across the composition domain, even if the branches do not run exactly parallel (compare the i-ii and iii-iv frequency gaps). In $Cd_{1-x}Be_xTe$ the effect of the dispersion achieves maximum at $x\sim1$, as emphasized by a long vertical arrow in Fig. 2a, to such extent that it overwhelms the effect of the local strain. This leads to an inversion of the Be-Te doublet at $x\sim1$, preceded by the crossing of Be-Te sub-branches at $x\sim0.8$, an unprecedented case among all re-examined alloys within the PM so far.

Generally, the large negative phonon shift generated by the Be-Te dispersion effect in $Cd_{1-x}Be_xTe$ at $x\sim1$ reflects how drastically the impurity Be-Te mode is impacted at large Be content by the local lattice distortions due to the Cd ↔ Be atom substitution at large Be content. The impact is comparatively less at $x\sim0$. Different lengths of arrows representing the dispersion-induced phonon shifts of Be-Te impurity modes at $x\sim0$ and ~1 in Fig. 2a are illustrative with this respect. Remarkably, the Be-Te dispersion effect does not generate an inversion of the Be-Te doublet in $Zn_{1-x}Be_xTe$[28] at $x\sim1$. Hence, the dispersion effect is less pronounced in $Zn_{1-x}Be_xTe$ than in $Cd_{1-x}Be_xTe$. As such, $Zn_{1-x}Be_xTe$ constitutes an exception among examined alloys so far, including WMA's, in which the impurity modes were shown to suffer a maximum dispersion effect. The distorted percolation doublets of $ZnSe_{1-x}S_x$ and $Si_{1-x}Ge_x$ were discussed on this very basis[53]. The Ga-As impurity mode likewise experiences the same (maximal) dispersion effect whether taken in the lattice-matched $Ga_{1-x}Al_xAs$ ($\frac{\Delta l}{l}\sim8‰$) or lattice-mismatched $Ga_{1-x}In_xAs$ ($\frac{\Delta l}{l}\sim7\%$) alloys[S13].

II.2. $Cd_{31}Be_1Te_{32}$ (SIESTA code) – isolated-impurity motif

An isolated Be atom in CdTe forms short Be-Te bonds suffering a hydrostatic tensile strain from the host CdTe medium corresponding to a large bond length. The Be-Te bond elongation Δl with respect to the natural bond length in the pure BeTe crystal, l_0, generates a Be-Te impurity mode in CdTe, referred to as the impurity-i mode in the main text, at a lower frequency than the parent BeTe TO frequency ($\omega_{T,0}$). The shift in TO frequency squared, $\Delta\omega_T^2$, relates to the variation in bond length Δl via the relation[S13,S14],

$$\frac{\Delta\omega_T^2}{\omega_{T,0}^2} = -6\gamma_T \cdot \frac{\Delta l}{l_0}, \qquad (1)$$

that involves the Grüneisen parameter of the parent TO mode, given by[S15]

$$\gamma_T = \frac{B_0}{\omega_{T,0}} \cdot \left(\frac{d\omega_T}{dP}\right)_{P=0}, \quad (2)$$

where P is the hydrostatic pressure and B_0 the bulk modulus.

Ab initio (SIESTA) calculations of the lattice relaxation and of the Γ-projected phonon density of states, that assimilates with the TO Raman spectrum, done on a large 2×2×2 (64-atom) fully relaxed cubic BeTe supercell yield l_0, B_0 and $\omega_{T,0}$ values of 2.406 Å, 63.7 GPa and 478 cm^{-1} at ambient pressure. The latter values nearly match the AIMPRO ones reported in Figs. 1d and 2a. The *ab initio* insight further predicts a linear increase of the TO frequency with pressure in the range 0– 20 GPa at the rate of ~10.05 cm^{-1} *per* GPa. The resulting γ_T estimate via Eq. (2) for the parent BeTe TO mode is ~1.33.

Similar SIESTA calculations done at 0 GPa on a similar zincblende-type $Cd_{31}Be_1Te_{32}$ supercell containing a unique Be atom give the Be-Te impurity-i mode at ~414 cm^{-1}. This emerges slightly above the experimental TO_{Be-Te}^{Cd} one at x~0 (Fig. 2a), corresponding to a Be-Te elongation of Δl~0.063 Å with respect to the natural-bulk l_0 value, specified above. The obtained *ab initio* frequency exceeds by ~10 cm^{-1} the impurity-i one derived by solely taking into account the effect of local strain, given by Eq. (1). The difference is due to the negative dispersion of the BeTe TO mode, impacting all i-to-iii impurity modes in principle[53], as indicated by vertical arrows in Fig. 2a. Such study of the lattice relaxation and of the lattice dynamics around an isolated Be impurity in CdTe (x~0), consistently conducted end-to-end within an *ab initio* (SIESTA) approach, emphasizes the important role of the phonon dispersion besides the local strain in shaping the Be-Te percolation doublet of $Cd_{1-x}Be_xTe$.

II.3. $Cd_{106}Be_2Te_{108}$ and $Cd_2Be_{108}Te_{108}$ (AIMPRO code) – Cd-Te signal vs. paired-impurity motifs

Fig. S6 displays high-pressure *ab initio* (AIMPRO) CdTe-like TO Raman spectra obtained by using large (216-atom) fully-relaxed cubic $Cd_{106}Be_2Te_{108}$ and $Cd_2Be_{108}Te_{108}$ supercells containing similar Be and Cd impurity-duos connected via Te. The respective pressure ranges are 0– 5 GPa (Fig. S6a) and 0 – 20 GPa (Fig. S6b). Such data come in support of the discussion of the pressure dependence of the Cd-Te doublet of $Cd_{1-x}Be_xTe$ done in the main text.

The Cd-Te doublet exhibits nearly the same spacing between the relevant iii-iv (x~0) and i-ii (x~1) Cd-Te modes at both ends of the composition domain. The duo-impurity modes are identified via their wavevectors, depending on whether they point along the duo, in reference to the in-chain mode noted ii and symbolized by ↔, or perpendicular to it, corresponding to out-of-chain modes regouped under the label i and marked ↮, yielding five possible variants[28]. For the host bond species, the distinction between "alien" and "same" environments is established through the Raman intensity. This is small for the close-duo mode next to the impurity motif, noted iv, representing the "alien" case. Oppositely, the Raman intensity is large for the bulk mode that vibrates away from the impurity motif, noted iii, representing the "same" case. Under pressure the doublets either cross, as observed with $Cd_{106}Be_2Te_{108}$ (Fig. S6a), or freeze into a phonon exceptional point at the resonance, as observed with $Cd_2Be_{106}Te_{108}$ (Fig. S6b). In fact, the exceptional point is already achieved at 0 GPa in the latter case. Note that the frozen submode, spotted by an arrow in (Fig. S6b), can be identified only by its wavevector, because its Raman intensity is zero.

II.4. $Cd_{54}Be_{54}Te_{108}$ (AIMPRO code) – intermediary composition

Fig. S7 reports on *ab initio* (AIMPRO) calculations of the bond angle distribution depending on pressure within the large disordered zincblende-type $Cd_{54}Be_{54}Te_{108}$ (x=0.5) supercell used to generate the corresponding *ab initio* (AIMPRO) Raman spectra shown in Fig. 2d. The data reveal a prohibitive supercell distortion from 15 GPa onwards. This is manifested by a deviation of bond angles from the nominal zincblende value of 109°, so that two distinct groups of bond angles are identified, as indicated by dotted lines in Fig. S7. The zincblende structure, increasingly damped with pressure, is only preserved up to 10 GPa. This fixes the limit for the $Cd_{54}Be_{54}Te_{108}$ *ab initio* Raman study reported in Fig. 2d. A similar collapse is suffered from 10 GPa onwards by the CdTe-like $Cd_{106}Be_2Te_{216}$ supercell used to provide an *ab initio* (AIMPRO) insight into the pressure dependence of the $Cd_{1-x}Be_xTe$ Raman spectra in the Be-dilute limit (x~0, Fig. 2c).

III. $Cd_{1-x}Be_xTe$ lattice dynamics – Raman scattering

III.1. Experimental Raman study at ambient pressure (x ≤0.11)

Fig. S8 displays a selection of unpolarized $Cd_{1-x}Be_xTe$ (x ≤0.11) Raman spectra taken at ambient pressure in the backscattering geometry on non-oriented crystal faces using the red (632.8 nm, for x=0 and 0.05) and blue (488.0 nm, for x=0.11) laser lines, near-resonant with E_0 and $E_0+\Delta_0$, respectively (Fig. 1b). The Raman signal consists of a quasi-degenerated TO-LO Be-Te impurity mode situated at much higher frequency than the CdTe-lattice TO-LO band, *i.e.*, 140 – 170 cm^{-1}. Near-resonance conditions favor the polar LO's compared with the non-polar TO's via the Fröhlich mechanism[S16,S17]. The LO emphasis is especially pronounced with the red laser excitation, manifested by the strong emergence of the second-order matrix Raman signal, noted 2× LO_{Cd-Te}. A pure Be-Te TO insight is searched for at the largest available Be content (x=0.11) by exciting a (110)-cleaved crystal face at normal incidence with the less resonant blue laser line and collecting the scattered light in backscattering. This corresponds to a (TO-allowed, LO-forbidden) scattering geometry[S17].

The Raman spectrum at minimal Be content, *i.e.*, 5 at.%, provides a crude experimental estimate of the Be-Te impurity-i frequency in CdTe, *i.e.*, ~390 cm^{-1}. This is in close agreement with existing data in the literature[49,50]. Besides, the comparison between the LO-like and TO-like $Cd_{0.89}Be_{0.11}Te$ Raman spectra, respectively obtained on non-oriented and (110)-cleaved crystal faces, resolves a finite Be-Te TO-LO splitting emphasized by dotted lines in Fig. S7. By decreasing x the lattice constant a increases (Fig. S1b). This distances cations and anions and hence softens the chemical bonds. Consequently, the unpolar-TO Raman frequencies, that scale as the square roots of the effective bond force constant[26], are downward shifted. The polar LO Raman frequencies "mechanically follow" by converging progressively towards the non-polar TO's as the related bond fraction decreases. Eventually, the TO-LO degeneracy is achieved in the dilute limits, *i.e.*, x~0 for Be-Te and x~1 for Cd-Te. An overview of the experimental $Cd_{1-x}Be_xTe$ Raman frequencies is given in Fig. 2a (symbols).

III.2. Contour modeling of (high-pressure) TO Raman spectra – linear dielectric function approach

III.2.a. Ambient pressure

An overview of the TO $Cd_{1-x}Be_xTe$ Raman frequencies (curves) and intensities (thickness of curves) calculated throughout the composition domain at 0 GPa in absence of mechanical coupling between TO oscillators is given in Fig. 2a. At 0 GPa, the non-polar TO's are presumably decoupled and hence are explicitly assigned by specifying both the bond vibration and the local environment, via a subscript and a superscript, respectively, *i.e.*, $\{TO^{Be}_{Cd-Te}, TO^{Cd}_{Cd-Te}, TO^{Be}_{Be-Te}, TO^{Cd}_{Be-Te}\}$.

The uncoupled modes of Fig. 2a. are modeled by assimilating the TO Raman cross section with the imaginary part of the relative dielectric function ε_r, that captures the $\varepsilon_r \to \infty$ divergence characteristic of a purely-mechanical TO[47,S18]. The adopted four-mode $\{2\times(Cd-Te), 2\times(Be-Te)\}$ percolation-type description of $Cd_{1-x}Be_xTe$ conforms with the current *ab initio* findings, referring to the end i-to-iv modes. A sensitivity of both bond vibrations to their local CdTe- and BeTe-like environments at the first-neighbor scale is assumed by analogy with $Zn_{1-x}Be_xTe$[28]. This directly governs the Raman intensities. The mechanical bond force constants are linearly interpolated between the parent and impurity *ab initio* values. This leads to quadratic x-dependencies of the TO frequencies. A minimal broadening of 1 cm^{-1} is uniformly used for a clear resolution of neighboring features and for direct comparison of the Raman intensities, as apparent via the color code. The remaining input parameters are determined *ab initio* (Sec. SIII.2); no adjustable parameters are used.

More precisely, a classical form is used for $\varepsilon_r(\omega,x)$ including a linear background electronic contribution $\varepsilon_\infty(x)$ at high-visible frequencies besides the far-infrared phonon one. The latter is divided between four oscillators in total (p=1 to 4) modeled as p-Lorentzian functions. In each Lorentzian, the numerator represents the available amount of oscillator strength per p-mode $S^0_p(x)$ that monitors the p-type TO Raman intensity. $S^0_p(x)$ scales as the p-type parent oscillator strength,

i.e., $S_p^0 = \varepsilon_{\infty,p} \cdot \Omega_p^2/\omega_{T,p}^2$ with $\Omega_p^2 = \omega_{L,p}^2 - \omega_{T,p}^2$, weighted by the p-oscillator fraction, given by the f_p-term below. The denominator, *i.e.*, $\omega_{T,p}^2(x) - \omega^2 - j\gamma_p\omega$, monitors the position of the TO p-resonance in its x-dependence, given by $\omega_{T,p}^2(x) = k_p(x)/\mu_p$. In the latter expression, the numerator and denominator refer to the effective mechanical bond force constant of oscillator-p and to the reduced atomic mass of the p-bond, respectively. The phonon damping γ_p, introduced via a friction force, fixes the full width at half maximum of the p-type Raman peaks, *i.e.*, 1 cm^{-1} in the present case, as mentioned above. We assume linear $k_p(x)$ vs. x variations, in the spirit of the historical modified-random-element-isodisplacement (MREI, 1-bond→1-mode) model of Raman spectra for semiconductor alloys[26]. This generates quadratic $\omega_{T,p}^2(x)$ variations. By analogy with $Zn_{1-x}Be_x$-chalcogenides[25,28], a sensitivity of Be-Te vibrations to crystalline environment limited to nearest neighbors is considered. The corresponding 1D-oscillators behind the four TO's specified in brackets above can be casted as $\{Te(Cd-Te)Be, Te(Cd-Te)Cd, Te(Be-Te)Be, Te(Be-Te)Cd\}$, participating with weights $\{f_{Cd-Te}^{Be} = x \cdot (1-x), f_{Cd-Te}^{Cd} = (1-x)^2, f_{Be-Te}^{Be} = x^2, f_{Be-Te}^{Cd} = x \cdot (1-x)\}$. The TO Raman intensities, apparent via the color code in Fig. 2a, scale accordingly. Hence the two TO submodes forming a given (Cd-Te or Be-Te) doublet exhibit comparable Raman intensities at x~0.5.

Altogether, this leaves ten input parameters in total, out of which six relate to the parent compounds ($\varepsilon_{\infty,p}$,Ω_p,$\omega_{T,p}$) and four to the alloy taken in its (Cd, Be)-dilute limits. All parameters are determined *ab initio*, without leaving any adjustable one. The input frequencies, two per TO branch, are derived by implementing a simple *ab initio* (AIMPRO) protocol onto impurity Cd-duo and Zn-duo motifs, in reference to the i, ii and iv end frequencies in Fig. 2a. The parent $\omega_{T,p}$ bulk TO frequencies, symbolized *iii* in Fig. 2a, come out as by-products. The parent TO-LO splittings together with the parent $\varepsilon_{\infty,p}$ values, needed to estimate the parent oscillator strengths, are obtained by resorting to QE (see methods).

III.2.b. High pressure

Fair phenomenological modeling of the pressure-induced interplay between Raman intensities taking place within the relaxed-inverted Be-Te percolation doublet of $Cd_{1-x}Be_xTe$ at x~1 (Fig. 2f), apparent in *ab initio* data (Fig. 2e), is achieved within a linear dielectric approach by assuming a weak mechanical coupling. The characteristic frequency ω'=50 cm^{-1}, similar to that used with ZnBeTe[28], is scaled down by roughly an order of magnitude with respect to the TO frequencies of the raw-uncoupled (TO_{Be-Te}^{Cd}, TO_{Be-Te}^{Be}) oscillators, emerging close to ~480 cm^{-1}. The corresponding pressure dependencies are represented by straight and dotted lines in Fig. 2f. A relevant expression for the corresponding Raman cross section has lately been derived in Ref.[28], given specifically by Eq. (7) therein. A simplified form is currently used in which the coupling term at the numerator is disregarded compared with the two main terms standing for the raw-uncoupled oscillators. For better visualization of trends, the dielectric study has been moved from dilute (x~1) to minor (x=0.81) Cd content, further offering a direct comparison with the regular $Zn_{1-x}Be_xTe$ case, referring to Fig. 1c of Ref.[28]. The peak frequencies are artificially preserved as such. A minimal phonon damping of 1 cm^{-1} is uniformly taken, for clarity.

The small coupling successfully mimics the minimal, but finite, *ab initio* splitting that manifests the anticrossing of the coupled (TO_{Be-Te}^{-}, TO_{Be-Te}^{+}) near the resonance, corresponding to frequency matching of the raw-uncoupled oscillators. The magnitude of the anticrossing near the resonance, emphasized by paired arrows in Fig. 2f, is less than ~3 cm^{-1}. The mechanical coupling achieves maximum at the resonance, occurring around 20 GPa, where the two Be-Te TO submodes exhibit comparable Raman intensities. A further pressure increase to 30 GPa eventually results in a proper doublet inversion (Fig. 2f), as the oscillators tend to decouple by shifting away from the resonance.

Supplementary Information-only References

[S1] Wronkowska, A. A. *et al.* Optical spectra of $Zn_{1-x}Be_xTe$ mixed crystals determined by IR-VIS-UV ellipsometry and photoluminescence measurements. *Thin Solid Films* **519,** 2795–2800 (2011).

[S2] Postnikov, A. V., Pagès, O. & Hugel, J. Lattice dynamics of the mixed semiconductors (Be,Zn)Se from first-principles calculations. *Phys. Rev. B* **71,** 115206 (2005).

[S3] Ganguli, T. *et al.* Lattice relaxation in the highly-contrasted $Zn_{1-x}Be_xSe$ alloy: An extended x-ray absorption fine structure study. *J. Appl. Phys.* **108,** 083539 (2010).

[S4] Eckner, S. & Schnohr, C. S. Private communication.

[S5] Proffen, Th., Billingue, S.J.L., Egami, T. & Louca D. Structural analysis of complex materials using the atomic pair distribution function – a practical guide. *Z. Kristallogr.* **218,** 132–143 (2003).

[S6] Qiu, X. *et al.* Reciprocal-space instrumental effects on the real-space neutron atomic pair distribution function. *J. Appl. Cryst.* **37,** 110–116 (2004).

[S7] Boyce, J. B. & Mikkelsen, Jr., J. C. Local structure of pseudobinary semiconductor alloys: An X-ray absorption dine structure study. *J. Cryst. Growth* **98,** 37–43 (1989).

[S8] Mikkelsen, Jr., J. C. & Boyce, J. B. Atomic-scale structure of random solid solutions: Extended X-ray-absorption-fine-structure study of $Ga_{1-x}In_xAs$. *Phys. Rev. Lett.* **49,** 1412–1415 (1982).

[S9] Pellicer-Pores, J. *et al.* X-ray-absorption fine-structure study of $ZnSe_{1-x}Te_x$ alloys. *J. Appl. Phys.* **96,** 1491–1498 (2004).

[S10] Pagès, O. *et al.* Percolation picture for long wave phonons in zincblende mixed crystals: from (Zn,Be)-chalcogenides to (Ga,In)As. *J. Phys. Condens. Matt.* **18,** 577–592 (2006).

[S11] Schnohr, C.S. Compound semiconductor alloys from atomic scale structure to bandgap bowing. *Appl. Phys. Rev.* **2,** 031304 (2015).

[S12] Verleur, H.W. & Barker, A.S, Jr. Infrared lattice vibrations in $GaAs_yP_{1-y}$ alloys. *Phys. Rev.* **149,** 715-729 (1966).

[S13] Groenen, J. *et al.* Optical-phonon behavior in $Ga_{1-x}In_xAs$: The role of microscopic strains and ionic plasmon coupling. *Phys. Rev. B* **58,** 10452–10462 (1998).

[S14] Pagès, O., Chafi, A., Fristot, D. & Postnikov, A. V. (Ga,In)P: A standard alloy in the classification of phonon mode behavior. *Phys. Rev. B* **72,** 165206 (2006).

[S15] Kuball, M. *et al.* Raman scattering studies on single-crystalline bulk AlN: temperature and pressure dependence of the AlN phonon modes. *J. Cryst. Growth* **231**, 391–396 (2001).

[S16] Geurts, J. Analysis of band bending at III-V semiconductor interfaces by Raman spectroscopy. *Surf. Sci. Rep.* **18,** 1-90 (1993).

[S17] Yu, P.Y. & Cardona, M. Fundamentals of Semiconductors, 4th ed. (Springer-Verlag, Heidelberg, 2010), Chap. 7, pp. 345-426.

[S18] Born, M. & Huang, K. Dynamical Theory of Crystal Lattice (Oxford University press, 1954), Chap. 2, pp. 38-128.

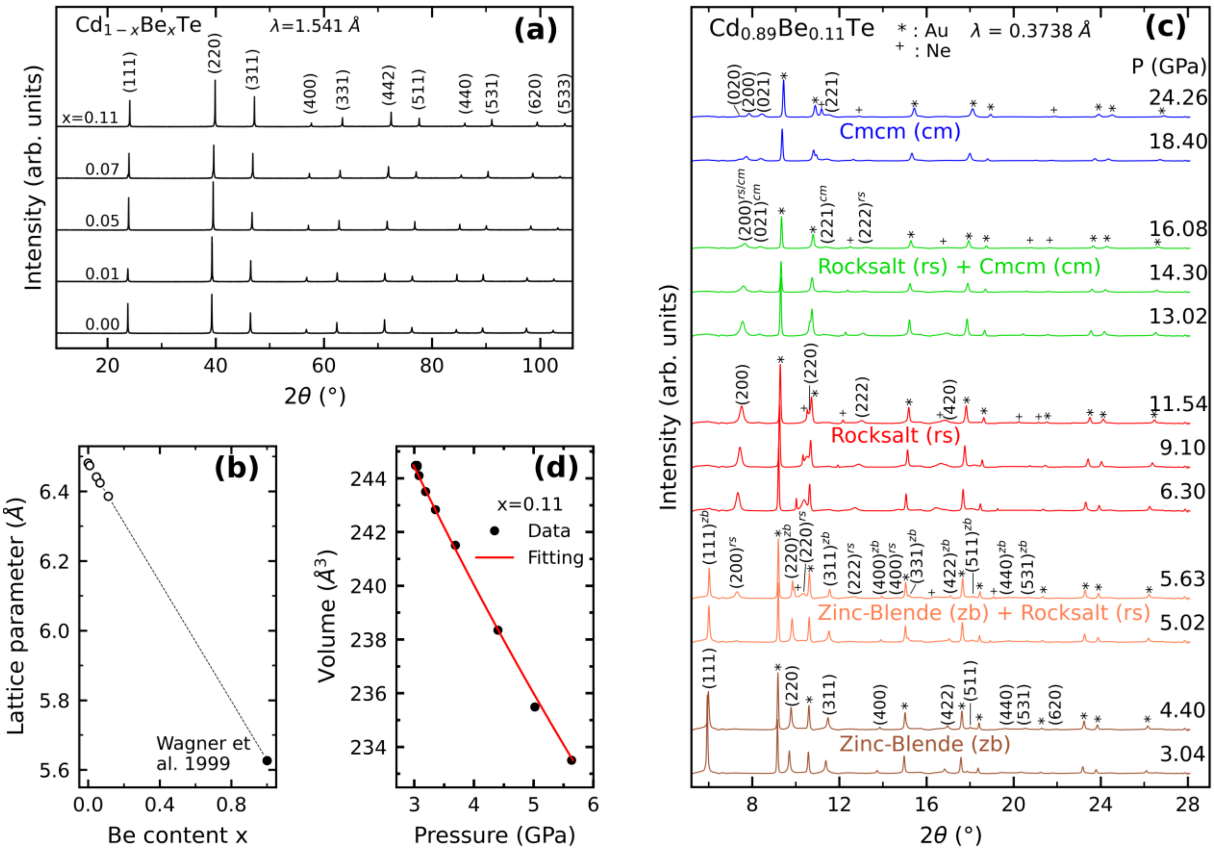

Figure S1 │ (High-pressure) $Cd_{1-x}Be_xTe$ ($x \leq 0.11$) X-ray diffraction data. (a) Powder $Cd_{1-x}Be_xTe$ X-ray diffractograms obtained at ambient pressure in laboratory. **(b)** Corresponding x-dependence of the lattice parameter. The BeTe value, taken from the literature[35], is added to complete the trend. The linearity is emphasized (dotted line). **(c)** selection of $Cd_{0.89}Be_{0.11}Te$-powder X-ray diffractograms obtained at increasing pressure. The individual peaks are labelled via the (hkl) Miller indices of the corresponding diffraction planes in various structural phases, as indicated. Additional diffraction peaks originate from Au and Ne used for pressure calibration and as the pressure transmitting medium, respectively. **(d)** Corresponding pressure dependence of the unit cell volume fitted to the Birch-Murnaghan equation of state.

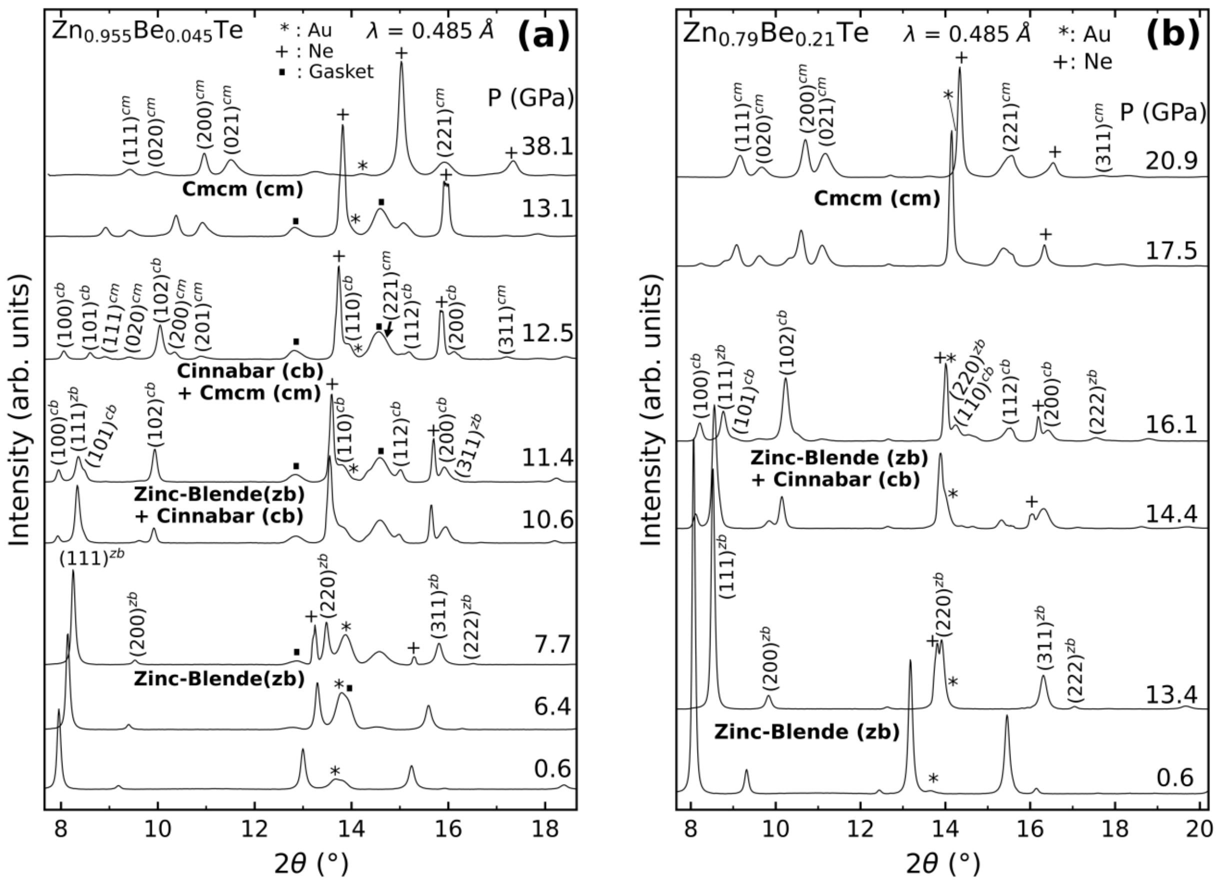

Figure S2 │ **High-pressure $Zn_{1-x}Be_xTe$ X-ray diffraction data at moderate Be content.** Selection of **(a)** $Zn_{0.955}Be_{0.045}Te$ and **(b)** $Zn_{0.79}Be_{0.21}Te$ powder X-ray diffractograms obtained at increasing pressure. The individual peaks are labelled via the (hkl) Miller indices of the corresponding diffraction planes in various structural phases, as indicated. Additional diffraction peaks originate from Au and Ne used for pressure calibration and as the pressure transmitting medium, respectively, and from the gasket, as specified.

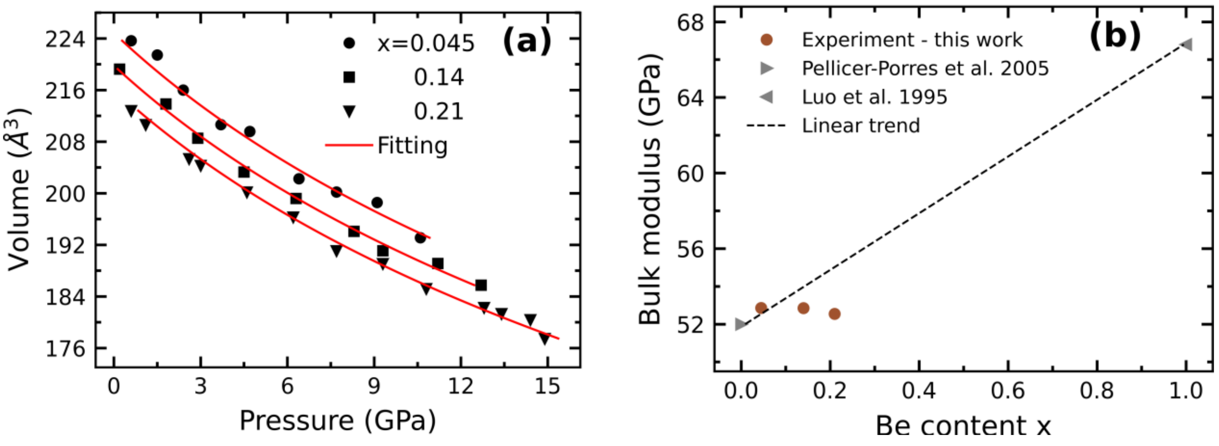

Figure S3│ $Zn_{1-x}Be_xTe$ bulk modulus. (a) Pressure dependencies of the unit cell volume derived from the high-pressure $Zn_{1-x}Be_xTe$ X-ray diffractograms (partially reported in Fig. S2) and fitted to the Birch-Murnaghan equation of state. similar data taken with $Zn_{0.86}Be_{0.14}Te$ in the same run of experiment are added. **(b)** Resulting B_0 vs. x variations.

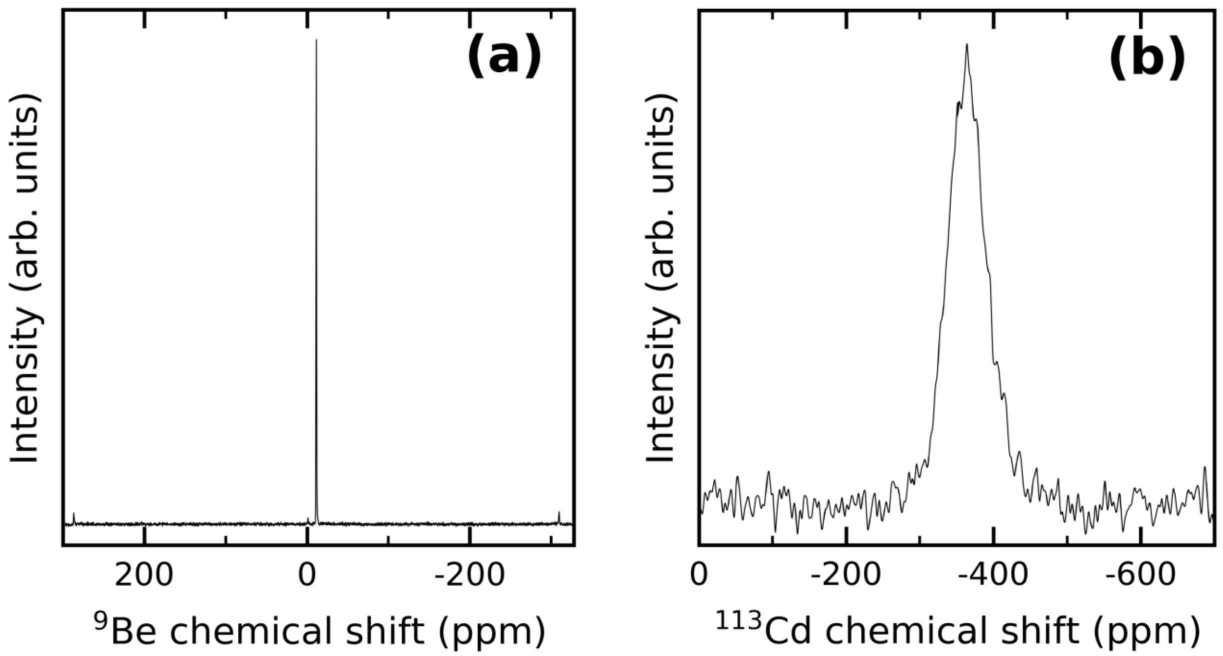

Figure S4 │ Substituent-related $Cd_{0.93}Be_{0.07}Te$ NMR spectra. (a) ^{9}Be and **(b)** ^{113}Cd NMR standard direct acquisition spectra.

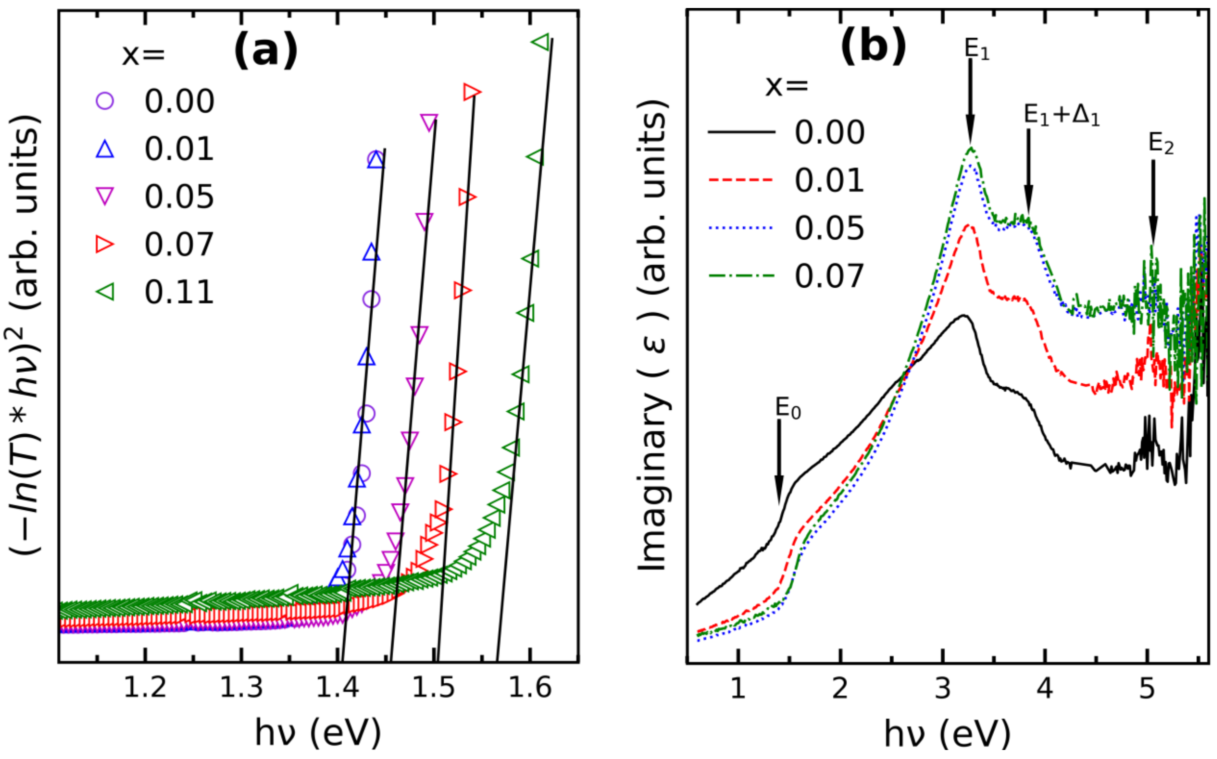

Figure S5 │ **$Cd_{1-x}Be_xTe$ transmission and ellipsometry data.** **(a)** $Cd_{1-x}Be_xTe$ Tauc plot (curves) of transmission data (symbols) giving access to E_0. **(b)** Corresponding ellipsometry data obtained by direct, *i.e.*, model free, wavelength-per-wavelength inversion of the sine and cosine of the depolarization angles measured by ellipsometry. The main electronic transitions are indicated.

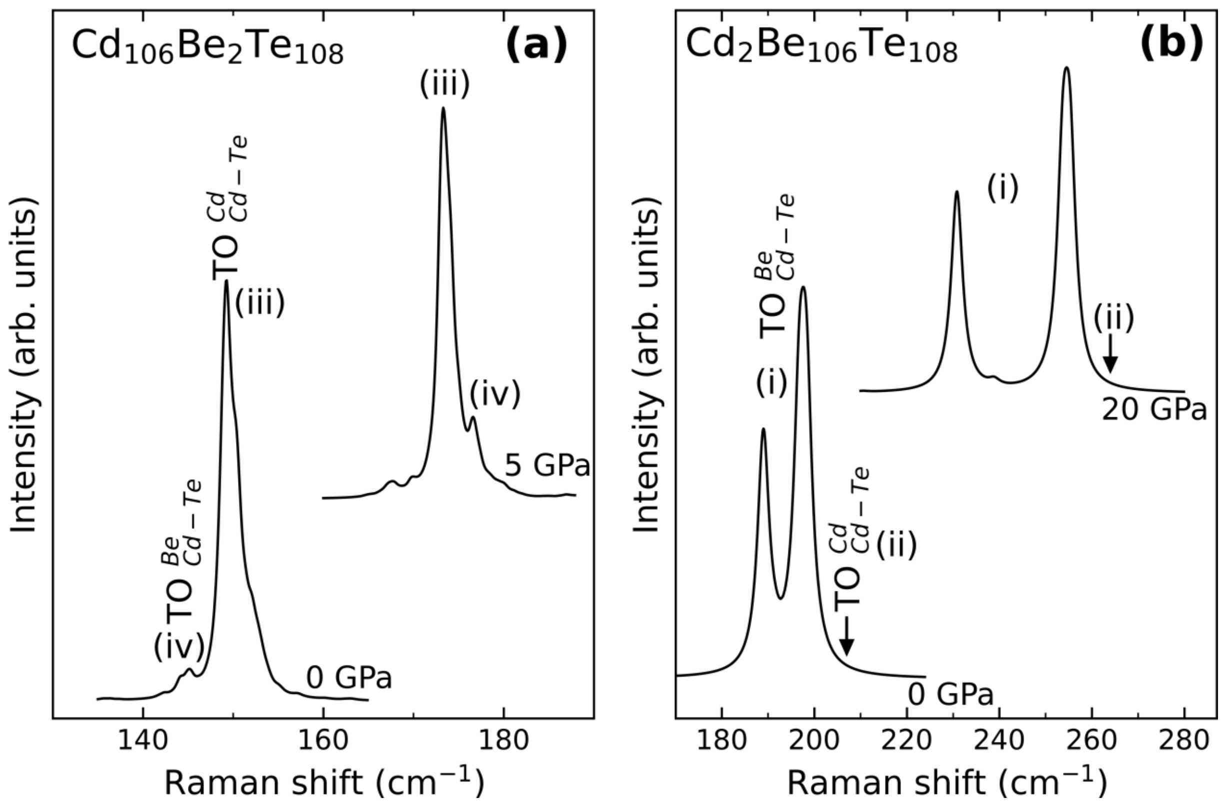

Figure S6 │ High-pressure *ab initio* (AIMPRO) CdTe-like Raman signals of $Cd_{1-x}Be_xTe$ generated by impurity-duos (x ~ 0,1). (a) $Cd_{106}Be_2Te_{108}$. **(b)** $Cd_2Be_{106}Te_{108}$. Under pressure the CdTe-like branches either cross **(a)** or freeze **(b)** at the resonance. The frozen submode, identified by its wavevector, is spotted by a vertical arrow.

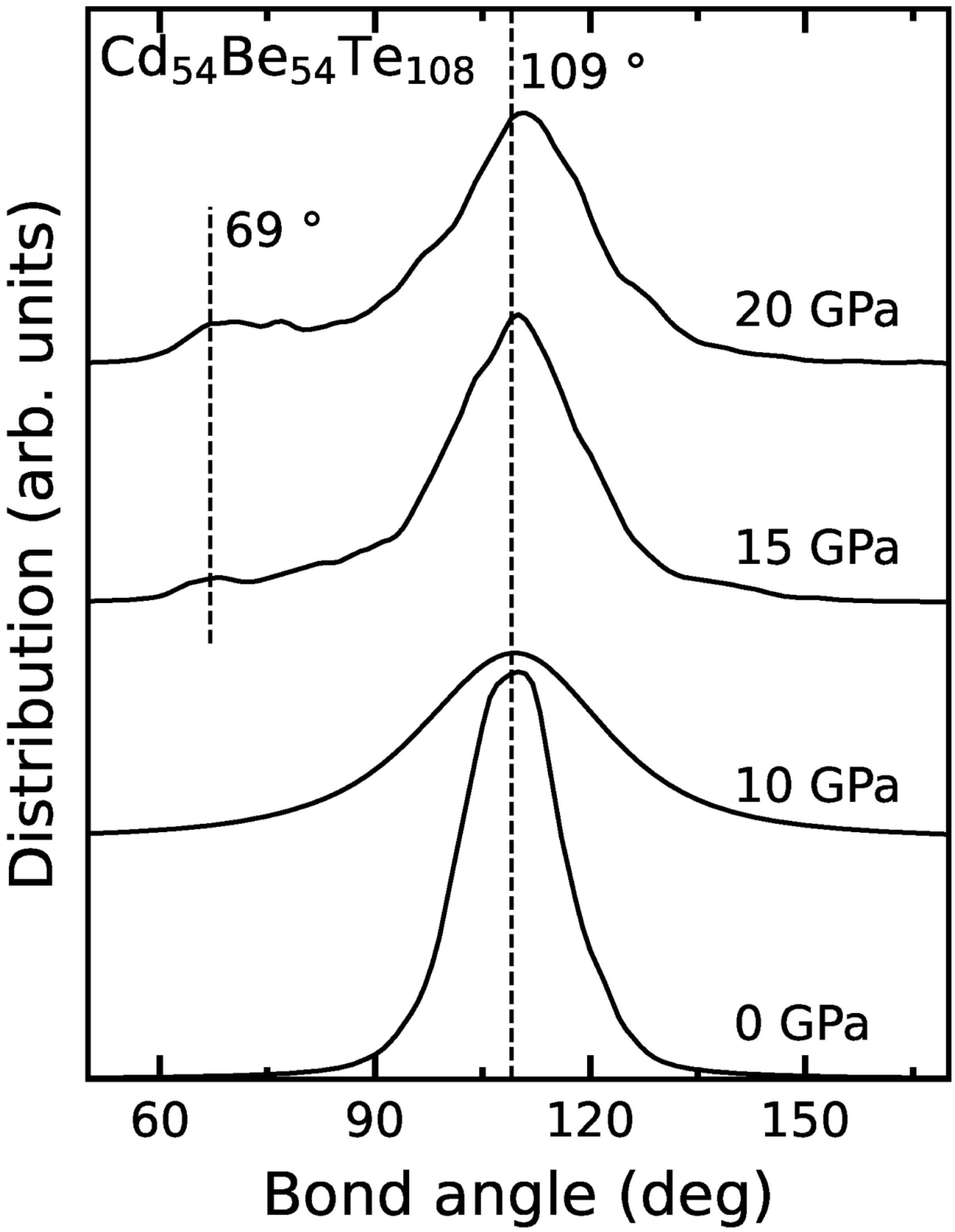

Figure S7 │ (High-pressure) *ab initio* (AIMPRO) insight into the $Cd_{54}Be_{54}Te_{108}$ lattice relaxation. Pressure dependence of the *ab initio* bond-angle distribution within the $Cd_{54}Be_{54}Te_{108}$ zincblende-type supercell optimized to a random Cd↔Be substitution bonds used to calculate the *ab initio* (AIMPRO) Raman spectra reported in Fig. 2d. The nominal angle value in the zincblende structure, *i.e.*, 109° (dotted line), is indicated, for reference purpose. A significant deviation down to 69° from 15 GPa onwards is emphasized (dotted line).

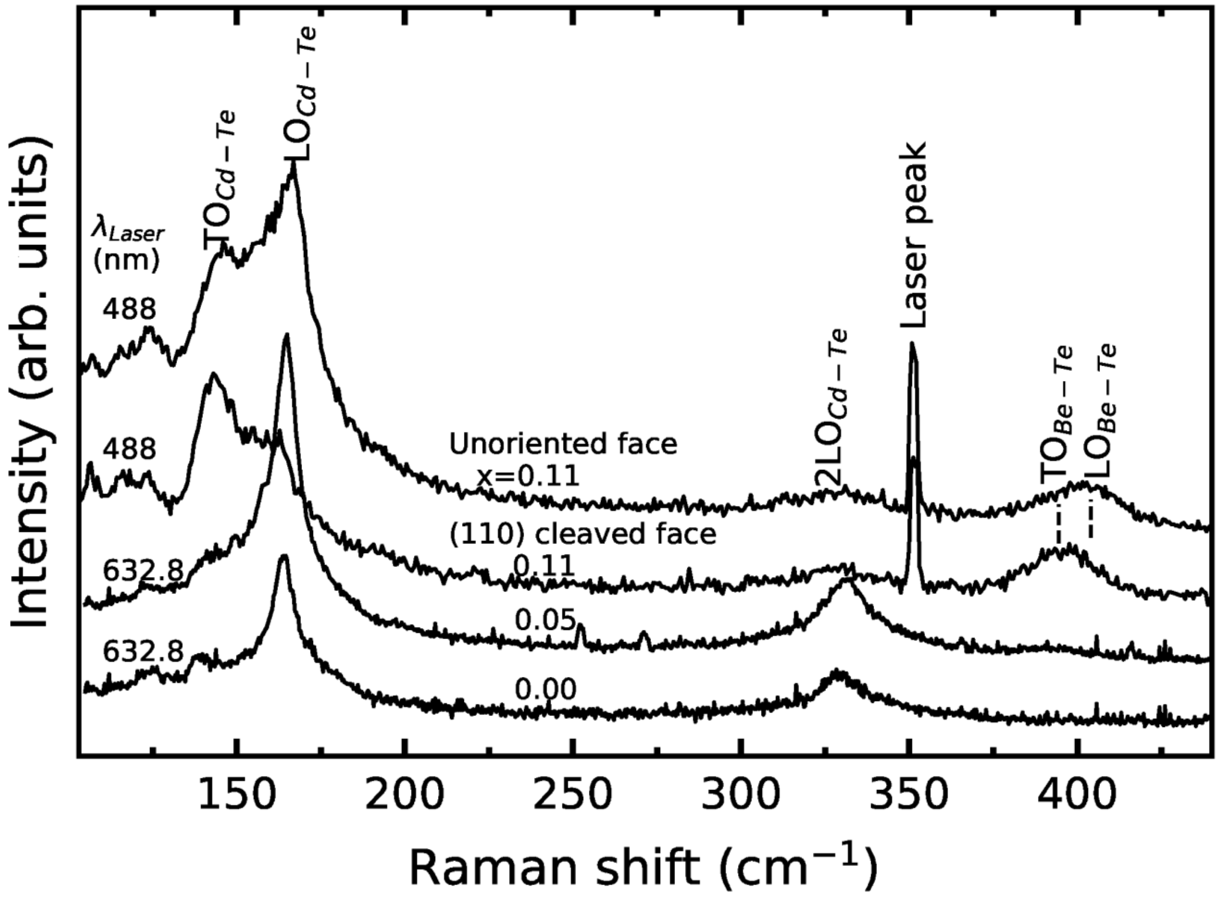

Figure S8│ Experimental $Cd_{1-x}Be_xTe$ (x ≤0.11) Raman spectra at ambient conditions. LO-like $Cd_{1-x}Be_xTe$ Raman spectra taken in the backscattering geometry on unoriented crystal faces polished to optical quality using the near-resonant red (632.8 nm) and blue (488.0 nm) laser lines. A pure-TO $Cd_{0.89}Be_{0.11}Te$ Raman spectrum taken with the 488.0 nm laser line in the backscattering geometry at normal incidence onto a (110)-cleaved face is added, for comparison. Paired dotted lines mark a finite TO-LO splitting at x=0.11.
